# Evaluation of linear *versus star*-like polymer anti-cancer nanomedicines in mouse models^☆^

**DOI:** 10.1016/j.jconrel.2022.11.060

**Published:** 2022-12-10

**Authors:** Libor Kostka, Lenka Kotrchová, Eva Randárová, Carolina A. Ferreira, Iva Malátová, Hye Jin Lee, Aeli P. Olson, Jonathan W. Engle, Marek Kovář, Weibo Cai, Milada Šírová, Tomáš Etrych

**Affiliations:** a Institute of Macromolecular Chemistry CAS, Department of Biomedical Polymers, Heyrovského nám. 2, Prague 6 16206, Czech Republic; b Department of Biomedical Engineering, University of Wisconsin-Madison, Madison, WI, United States; c Institute of Microbiology CAS, Laboratory of Tumor Immunology, Vídeňská 1083, Prague 4 14220, Czech Republic; d Department of Pharmaceutical Sciences, University of Wisconsin-Madison, Madison, WI, United States; e Department of Radiology and Medical Physics, University of Wisconsin-Madison, Madison, WI, United States

**Keywords:** Drug delivery, Cancer, Polymeric carriers, HPMA, Biodistribution, Positron emission tomography

## Abstract

Nanomedicines are considered next generation therapeutics with advanced therapeutic properties and reduced side effects. Herein, we introduce tailored linear and star-like water-soluble nanosystems as stimuli-sensitive nanomedicines for the treatment of solid tumors or hematological malignancies. The polymer carrier and drug pharmacokinetics were independently evaluated to elucidate the relationship between the nanosystem structure and its distribution in the body. Positron emission tomography and optical imaging demonstrated enhanced tumor accumulation of the polymer carriers in 4T1-bearing mice with increased tumor-to-blood and tumor-to-muscle ratios. Additionally, there was a significant accumulation of doxorubicin bound to various polymer carriers in EL4 tumors, as well as excellent *in vivo* therapeutic activity in EL4 lymphoma and moderate efficacy in 4T1 breast carcinoma. The linear nanomedicine showed at least comparable pharmacologic properties to the star-like nanomedicines regarding doxorubicin transport. Therefore, if multiple parameters are considered such as its optimized structure and simple and reproducible synthesis, this polymer carrier system is the most promising for further preclinical and clinical investigations.

## 1. Introduction

Despite the extensive development of nanotechnology-based therapeutic approaches for cancer treatment, only a limited number of nanosized therapeutics, especially polymer-drug conjugates, have been successfully introduced to clinical practice. [1] Unsuccessful clinical translation of polymer-drug conjugates with highly promising activity in animal models is caused by diverse aspects including the complexity of human tumors with often minimized enhanced retention and permeation (EPR) effect, increased drug-resistance compared to *in vivo* mouse models [2], numerous metastases with poor blood perfusion [3], unexpected immunogenicity or toxicity, laborious and expensive manufacturing, and poor synthesis reproducibility. [4,5] Therefore, the preclinical evaluation of newly developed nanosized therapeutics should involve the evaluation of aspects such as the distinct activity and toxicity in humans compared to animals and the synthetic process.

Much research has focused on the synthesis of tumor-targeted polymer conjugates based on *N*-(2-hydroxypropyl) methacrylamide (HPMA) copolymers with diverse structures (*e.g.*, linear, branched, dendritic, and micellar) to increase the hydrodynamic size and thus, enhance their accumulation in tumors. [6–8] Many of these polymer-drug conjugates exhibited surprisingly high anti-tumor efficiency in various tumor models, particularly those using small tumor nodules with very early treatment or with a pronounced EPR effect and homogenous cancer cell populations sensitive to various chemotherapeutics. [9] Their superior activity has not been proved in clinical trials, implying that the enhanced nanomedicine accumulation caused by the EPR effect may play only a supporting role in most human tumors but their overall toxicity has repeatedly proven to be considerably reduced in comparison to the corresponding free drug in animal models as well as in patients. [10,11]

The phenomenon of significantly reduced toxicity of the polymer-bound drugs compared to the corresponding free drugs poses a question of whether the anti-tumor activity of these systems should be compared using an equitoxic rather than equimolar dosage. In addition, by increasing the hydrodynamic size of the polymer conjugate, *i.e.*, branched, dendritic or micellar structures, renal excretion and consequently long-term toxicity is increased compared to the polymer conjugates smaller than the limit of the renal threshold. The appropriate size and design of the nanocarrier structure are key not only for the solid tumor accumulation but also for the extravasation and penetration into the tumor. [12] HPMA-based pirarubicin conjugates enhanced the penetration into the various cancer cell spheroids thus delivering the drug considerably deeper compared to pirarubicin alone in the 3D tumor model *in vitro.* However, almost identical penetration through spheroids for linear and star-like copolymers with sizes between 10 and 30 nm was observed. [13] Interestingly, linear–dendritic block copolymers based on amphiphilic poly[(ethylene glycol) methyl ether methacrylate] (POEGMA) linear-peptide dendritic prodrug (LDBCs) prepared by reversible addition-fragmentation chain transfer polymerization, were described as attractive candidates for smart drug-delivery of doxorubicin. [14]

Recent advances in controlled polymerization techniques and coupling chemistry have enabled the facile synthesis of complex polymer-based nanomedicines with a tailored structure, functionality, and very low dispersity. [15–17] Polymer carriers with various structures (linear, branched, dendritic, micelles, nanoparticles), sizes, and different stability upon application (stable, hydrolytically, or enzymatically degradable) can be designed. [18] Nevertheless, the formulation complexity and the extra care during the synthesis or storage needed for successful nanomedicine upscaling and product development must be considered.

Herein, we synthesized and compared five water-soluble nanosized carriers based on HPMA copolymer conjugates with the cytostatic drug doxorubicin. These conjugates with different polymer structures and biodegradability influencing their biodistribution and excretion were compared in a model of hematologic malignancy with pronounced EPR effect (EL4 T cell lymphoma) and a model of triple-negative breast carcinoma 4T1 with high metastatic potential.

## 2. Experimental section/methods

Sections 2.1 and 2.5–2.11 are listed in SI.

### 2.2. Synthesis of linear polymer precursors

The statistical linear copolymer poly(*N*-(2-hydroxypropyl) methacrylamide-*co*-*N*-[6-(2-methyl-acryloylamino)-hexanoyl]-hydrazine carboxylic acid *tert*-butyl ester) (HPMA-*co*-MaAhNHNH-Boc) was prepared by reversible addition-fragmentation chain transfer (RAFT) [19] copolymerization using CTA-AIBN and V-70 (the initiator). The polymer precursor was synthesized in a molar ratio of monomer:CTA:initiator of 580:2:1 in a mixture of *tert*-butanol/DMAc (90/10 v/v) in monomer molar concentration of 0.7 mol^−1^ at 30 °C for 72 h. Briefly, HPMA (2.05 g, 14.2 mmol) and MaAhNHNH-Boc (390 mg, 1.26 mmol) were dissolved in 20 mL of *t*-BuOH, CTA (11.14 mg, 0.054 mmol), and the initiator (8.36 mg, 0.027 mmol) was dissolved in 1.08 mL DMAc. Both solutions were bubbled with argon for 10 mins in a polymerization ampule and polymerization was performed at 30 °C for 72 h. The polymer precursor was precipitated into a mixture of acetone and diethyl ether (2/1) and precipitation from methanol. The polymer was filtered and dried under vacuum (yield 1.92 g, 79%). The trithiocarbonate end groups were removed by reaction with an excess of azo initiator: 1.92 g of polymer and AIBN (400 mg, 2.43 mmol) was dissolved in 17.2 mL DMAc and reacted at 80 °C for 3 h. The polymer precursor was precipitated into ethyl acetate, filtered, and vacuum dried (yield 1800 mg). The reactive polymer precursor was prepared by deprotection of hydrazide groups in water (100 °C, 40 min). [20] The semitelechelic linear polymer LP2 with thiazolidine-thiol end groups was synthesized using CTA-TT by the method described above in a molar ratio of 580:2:1 (M:CTA:I).

### 2.3. Synthesis of star-like polymer precursors

The star-like polymer precursors were synthesized as described previously. [21] Briefly, the star-like precursor SP2 was synthesized by the reaction of bisMPA (TMP-G3-ammonium) amino groups with the TT-end group of linear precursors (LP2) in the molar ratio of 1:8. BisMPA dendrimer (19.14 mg, 2.7 mmol) and DIPEA (25.4 mg, 196.4 mmol) were dissolved in 4.0 mL DMAc. After 40 min, LP2 (830 mg, 21.6 mmol) in 14.4 mL DMA was added and the mixture was stirred for 3 h at 25 °C. The reaction mixture was precipitated into ethyl acetate and vacuum dried. Unreacted amino groups on the dendritic core were blocked with the TT derivative of acetic acid, except for the DFO conjugate in which DFO was coupled to the unreacted amino groups on the core.

All synthesized star-like precursors were purified by SEC (Sephacryl S-300, 0.15 M NaCl, and desalted by Sephadex G-25, water) to remove unreacted linear polymers. Star polymer precursors with bisMPA cores were deprotected in water, and star precursors with PAMAM cores were deprotected in the presence of TFA. Free hydrazide groups were determined by the TNBSA assay (ε_500_ = 17,100 L mol^−1^ cm^−1^, borate buffer). [22]

### 2.4. Synthesis of polymer conjugates

The linear and *star*-like polymer conjugates containing doxorubicin bound by hydrazone bonds were synthesized by the reaction of hydrazide groups with keto groups of doxorubicin in the presence of acetic acid. For example, the star polymer **SP1** (1.0 g) and doxorubicin (DOX) (110 mg) were dissolved in 10 mL anhydrous methanol in 800 μL of ice-cold acetic acid. The mixture was reacted for 18 h at 25 °C in the dark and unreacted doxorubicin was removed by column chromatography (Sephadex LH-20, methanol). Purified polymer conjugates were concentrated using a rotary vacuum evaporator, precipitated into ethyl acetate, and vacuum dried.

*Star*-like conjugates containing Dy676 were synthesized by reacting Dy676-NHS with hydrazide groups on the polymer precursor in DMSO. Briefly, star polymer SP1 (100 mg) and Dy676-NHS (2 mg) were dissolved in 10 mL anhydrous DMSO and the mixture was reacted for 18 h at 25 °C in the dark. Unreacted dye was removed by column chromatography in methanol (Sephadex LH-20). The product was concentrated, precipitated into diethyl ether, and vacuum dried.

For polymer biodistribution studies, the star-like systems were labeled with deferoxamine (DFO) by reacting the isothiocyanate derivative of DFO with amines on the dendritic cores. Briefly, 75 mg SP1, 7.2 mg DFO-NCS, and 4 μL DIPEA were dissolved in 1.5 mL DMSO and reacted overnight at 25 °C. The free amino groups on the dendritic core were blocked with activated acetic acid and precipitated into acetone/diethyl ether (1/1), filtered, and vacuum dried. The DFO content was determined spectroscopically after reaction with FeCl_3_•6 H_2_O. The synthesis and characterization methods were described previously. [21] The samples were evaluated for their fluorescence intensity and stability before application, see Fig. S1.

## 3. Results and discussion

**Fig. 1.**
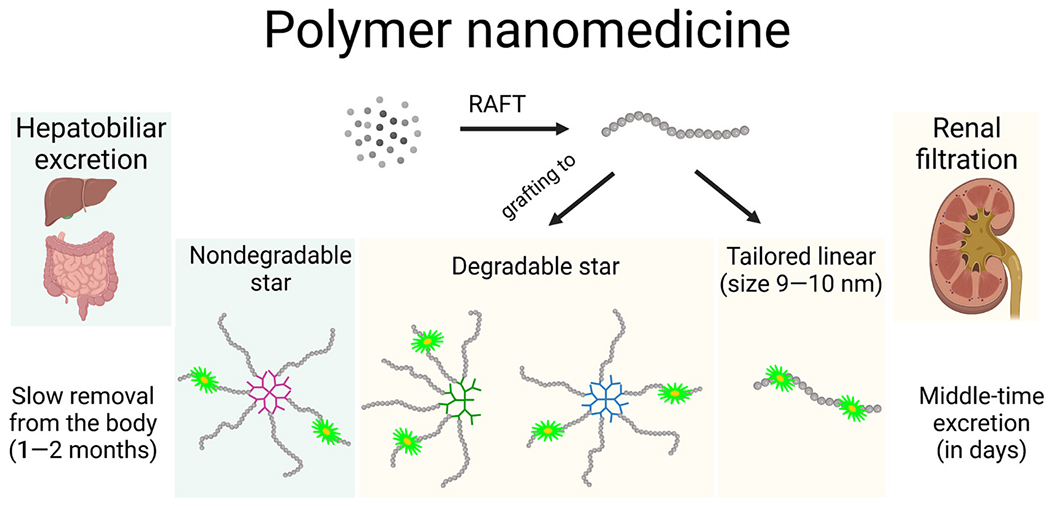
The overview of polymer nanomedicine used in this work from the chemical and excretion point of view.

Despite the promising results of novel targeted anti-cancer nanomedicines with potent anti-neoplastic effects and without any significant systemic toxicity in animal models, the same positive results have not been observed in clinical trials, hence very few nanosystems have been translated into clinical practice. [4] Nevertheless, these easily excreted nanomedicines with a relatively small nanoscale size ranging from 10 to 50 nm which allows for extended circulation, tumor accumulation due to the EPR effect, extravasation, and subsequent penetration even into avascular tumor parts are still ideal candidates for human cancer treatment. Herein, we aimed to compare the overall pre-clinical applicability of selected polymer-based nanomedicines based on the linear polymer tailored for prolonged circulation and tumor accumulation and *star*-like biodegradable water-soluble polymers which are highly efficient anti-cancer nanomedicines in different tumor models, see Fig. 1 for schematic description of studied systems. [8]

### 3.1. Polymer precursors and conjugates

Linear HPMA copolymers were synthesized by a controlled RAFT polymerization technique to produce monodispersed polymer systems. This technique allows the synthesis of copolymers with reactive groups not only along the main polymer chain (*i.e.*, hydrazide groups for drug attachment) but also at the end of the main chain end. Thus, linear copolymers were synthesized using functionalized or non-functionalized chain transfer agent CTA-TT/CTA-AIBN and azo initiator V-70 with low decomposition temperature. The polymer precursor LP1 was designed and synthesized as the precursor with a tailored structure and size close to the limit of renal filtration (50–70 kg/mol for HPMA-based linear copolymers) ensuring both the excretion by glomerular filtration, and prolonged circulation of the polymer-drug conjugate. The polymer precursor LP2 was synthesized to have a comparable length to LP1 with main chain-end reactive TT groups for subsequent star copolymer synthesis [23]. The characteristics of the polymer precursors with protected hydrazide groups are summarized in Table 1.

After the deprotection of hydrazide groups on LP1, doxorubicin was attached to the precursor *via* a pH-sensitive hydrazone bond which allows the release and re-activation of the anti-cancer activity of the drug within the tumor tissue. Importantly, the drug release occurs in the extracellular tumor space at pH 6.8–6.5 and is accelerated after the further pH decrease in tumor cells, namely in cell lysosomes. Drug attachment is not significantly affected by molecular weight or hydrodynamic size linear conjugates in aqueous solutions. The characteristics of the linear conjugate are summarized in Table 2.

*Star*-like water-soluble polymer systems are highly efficient drug delivery systems showing prolonged accumulation and enhanced accumulation in solid tumors. [24,25] However, their non-degradable structure and large size prevent renal elimination leading to extended body accumulation and considerably decreased maximum tolerated dose (MTD) compared to the corresponding linear conjugates easily excreted by the kidneys. [26] The overall toxicity of nanomedicines is crucial for further clinical translation and it is often overlooked when comparing diverse drug formulations. Therefore, we aimed to synthesize biodegradable star systems by grafting onto approach and compare their anti-cancer characteristics to a non-degradable star system and linear system with hydrodynamic size tailored to the limit of the renal threshold. Three biodegradable star systems were prepared either containing an amide bond or azide-alkyne click coupling between the polyester bisMPA core and polymer chains, see Fig. S2 and S3. The biodegradable star polymers differed in biodegradability rate, being higher for the amide-bond containing star polymers SP1 and SP2 (half-life of spontaneous hydrolysis around 26 days) and slower for SP3 containing the triazol-based linkage (half-life around 79 days), see Fig. S4. Importantly, the star polymers with a bisMPA core could be degraded by esterases in the tumor cells, thus their biodegradability could be even higher once the star systems are accumulated in solid tumors. All the star precursors were synthesized with a similar hydrodynamic size in the range of 21 to 24 nm to study the effect of the inner structure and biodegradability on therapeutic efficacy. The control non-degradable star polymer was synthesized using RAFT polymer precursors with low dispersity. The characteristics of synthesized star polymer precursors are summarized in Table 3.

The star-doxorubicin conjugates were prepared using the same procedure as for the linear conjugates and the star nanomedicines SC1 to SC4 were obtained using the stimuli-sensitive hydrazone bond for DOX attachment. The molecular weight, hydrodynamic size, and dispersity were not significantly affected by drug binding to the star precursors. All the conjugates contained around 10 wt% of the bound drug, see Table 4.

The linear and star-like precursors were labeled by fluorochrome Dy676 or iron chelator deferoxamine to determine the biodistribution. The content of both the dye and the chelator was sufficient for the following biodistribution study. Radiolabeling was performed at 37 °C (pH 7–8) for 2 h and yields were above 95% for all investigated compounds (Fig. S1). Characteristics of labeled polymers are summarized in Table 5.

### 3.2. Drug release and stability

Drug release from both the linear and star nanomedicines was studied in different pH buffer solutions (pH 7.4, 6.5, and 5.0) and there were no significant differences in the stability and release behavior of the polymer conjugates LC1, and SC1–4. [21] The polymer conjugates were relatively stable at pH 7.4 corresponding to bloodstream conditions and released up to 7% of the drug within 24 h. DOX release was accelerated when the pH dropped to 6.5 simulating the slightly acidified microenvironment within the tumor interstitium, with around 20% of DOX released within the same time. The highest DOX release was observed at pH 5 modeling the lysosomal compartment, where 70% was released within 5 h. In summary, all the polymer nanomedicines regardless of their internal structure showed favorable stimuli-sensitive behavior for tumor microenvironment-sensitive drug release.

### 3.3. Biodistribution studies

Since drug attachment modifies the pharmacokinetics (blood clearance) and tissue distribution of the polymer carrier, the evaluation of the biodistribution and pharmacokinetics of the advanced nanomedicines is key to predicting their activity and long-term toxicity. Such studies should precede the *in vivo* activity study to preselect the optimal nanomedicine, and thus, minimize the number of animals. Schematic representation of polymer systems used in this study is shown in Fig. S5. Three approaches were employed to validate the developed linear and star polymer nanosystems, positron emission tomography (PET) using ^89^Zr chelated with deferoxamine (DFO), optical imaging using the fluorescent dye Dy676, and the chemical analysis of tissue samples. Two tumor models were selected to reflect a different group of neoplastic diseases: i) EL4 T cell lymphoma was selected as a model of hematological malignancy characterized by a highly pronounced EPR effect; ii) 4T1 breast carcinoma was selected as a model of solid tumor characteristic with avascular tumor parts and progressively forming multiple metastases very early after the formation of a primary tumor.

#### 3.3.1. Biodistribution of polymer carriers

**Fig. 2.**
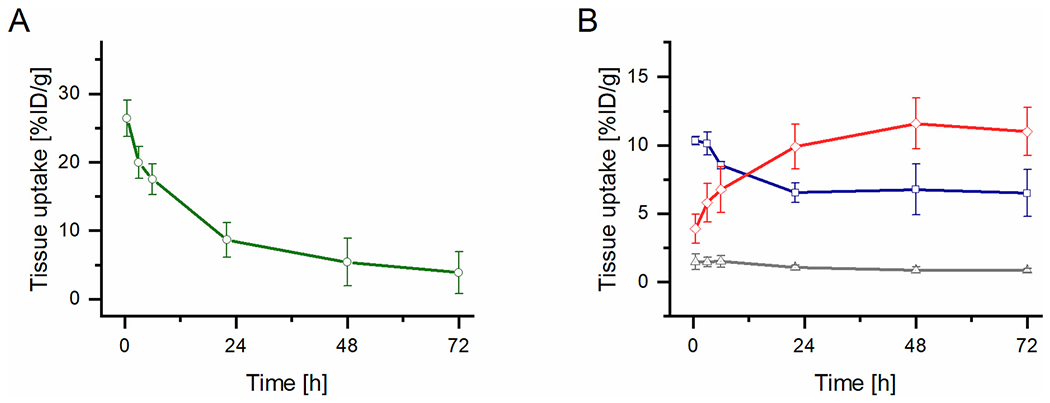
Time activity curves, calculated from PET ROI values, of ^89^Zr-LPet1 injected into the mice bearing 4T1 carcinoma in selected organs up to 72 h p.i., A: blood, B: tumor (red), liver (blue), muscle (grey), *n* = 3. (For interpretation of the references to color in this figure legend, the reader is referred to the web version of this article.)

The star polymer carriers with a biodegradable core were designed to maximize their tumor accumulation and to have low toxicity. Since we previously proved that the HPMA-based star drug conjugates with a non-degradable PAMAM dendrimer core have significantly enhanced tumor accumulation and higher toxicity *in vivo* in comparison to linear conjugates, it was anticipated that the modification of the dendrimer core enabling degradation and subsequent renal elimination would maintain the high tumor accumulation rate but decrease its toxicity. The results from two imaging methods, namely PET and optical imaging, were evaluated using 4T1 tumor-bearing mice to compare the potential of star carriers (SPet1, SPet2, SPet4, and SF1, SF2, SF4) with the linear carrier (LPet1 and LF1), with an optimized molar mass close to the renal elimination threshold, to improve drug pharmacokinetics. The linear polymer carrier (LPet1) rapidly accumulated within the tumor tissue and the relative content of injected dose per gram of tissue (%ID/g) peaked at 12% on the second-day post-injection (p.i.) and tumor accumulation remained high until the end of the experiment (Fig. 2). Tumor uptake was significantly higher than uptake in off-target tissues after 24 h p.i. Liver uptake corresponded well to the amount of the conjugate in the blood due to high liver perfusion and decreased concomitantly with blood clearance.

**Fig. 3.**
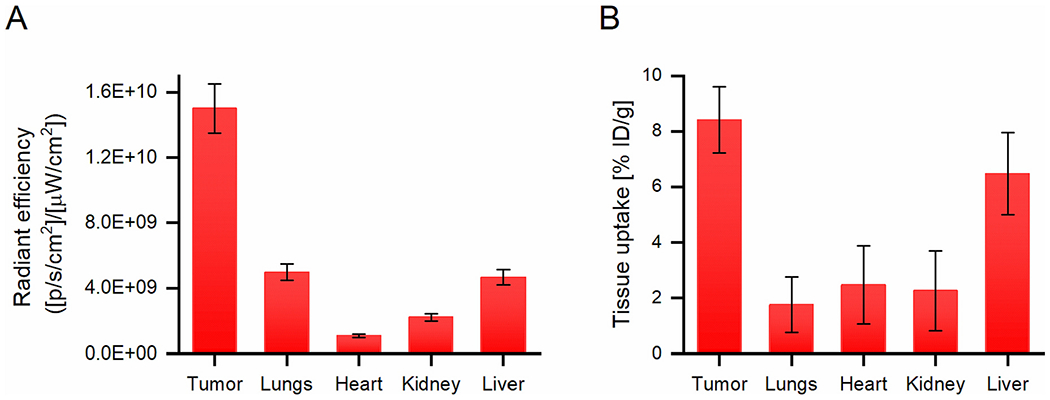
*Ex vivo* analysis of the tumor and other organs of mice bearing 4T1 carcinoma: A) Fluorescence Imaging: ROI analysis of selected organs 72 h p.i. of LF1 using total radiant efficiency [p/s/cm^2^/sr] / [μW/cm^2^]; B) Percentage of the ID/g of LPet1 of selected organs 72 h p.i., calculated from PET ROI values of LPet1, *n* = 3.

Serial *in vivo* optical images of 4T1 tumor-bearing mice confirmed the PET imaging results, with the linear LF1 polymer preferentially accumulating in the tumor tissue at 72 h p.i. (Fig. 3) and relatively low accumulation in healthy tissues. The fluorescence intensity *ex vivo* in the tumor was up to 10 times higher than in the heart and up to 3 times higher than in the liver and lungs after 72 h p.i. (Fig. 3A). Similarly, the *ex vivo* PET analysis of the tumors and healthy organs showed comparable results, with significantly higher tumor signal intensity in the lungs, heart, and kidney but only slightly lower in the liver (Fig. 3B). Taken together, it is evident that the linear copolymer accumulates significantly more in the tumor than in healthy tissues.

As expected, the increased hydrodynamic size led to prolonged blood circulation, thus, about 5% of ID was found in the blood 7 days p.i. in comparison to 2 days for the linear carrier. Furthermore, increased tumor accumulation was also confirmed.

**Fig. 4.**
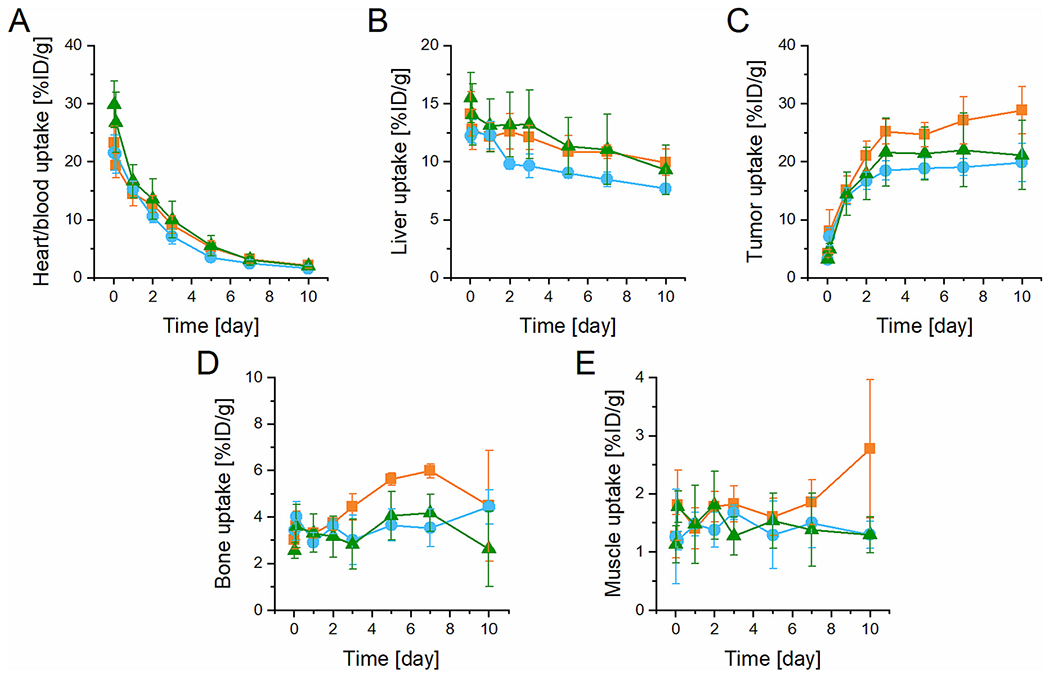
Quantitative region-of-interest (ROI) analysis. Time activity curves of A) heart/blood, B) liver, C) tumor, D) bone, and E) muscle upon i.v. injection of SPet1 (blue), SPet2 (green), or SPet4 (orange) into 4T1 tumor-bearing mice (*n* = 4). (For interpretation of the references to color in this figure legend, the reader is referred to the web version of this article.)

Despite the different structures and biodegradability rate of star polymer carriers, analogous biodistribution and tissue accumulation were obtained for all three *star*-like polymer conjugates regardless of their internal structure (Fig. S4 and Fig. S6). The highest uptake was in the tumor that plateaued 3–10 days p.i. with 19, 21, and 25 % ID/g for SPet1, SPet2, and SPet4, respectively (Fig. 4). The nondegradable star polymer SPet4 exhibited slightly higher accumulation in the tumor, bone, and muscle but this did not reach statistical significance compared to other star polymers. This is in line with the biodegradability study and supports our premise that SPet1 and SPet2 are degraded to smaller polymer chains which are eliminated *via* glomerular filtration. In contrast, the nondegradable SPet4 was slowly eliminated, probably *via* the hepato-biliary route, which may cause long-term persistence in the body and increased toxicity.

**Fig. 5.**
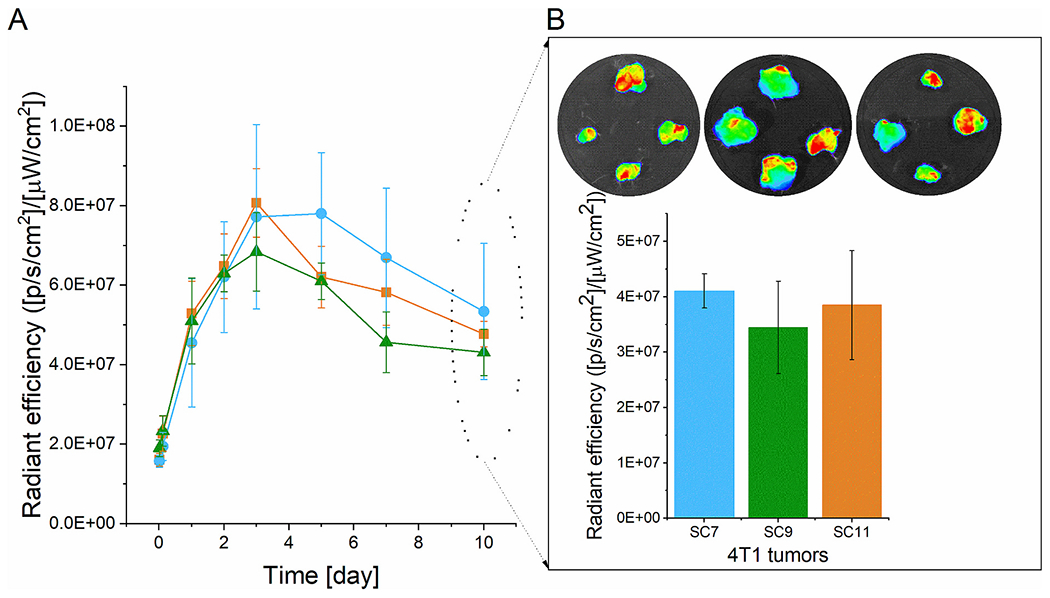
A) Time activity curves of the tumor calculated from ROI analysis of fluorescence imaging obtained from mice bearing 4T1 mouse carcinoma injected with SF1 (blue), SF2 (green), and SF4 (orange) (*n* = 4). B) *Ex vivo* images of tumors 10 days p.i. of the tracers. ROI analysis calculated from *ex vivo* images of activity retained in the tumor at day 10 p.i. (n = 4). (For interpretation of the references to colour in this figure legend, the reader is referred to the web version of this article.)

**Fig. 6.**
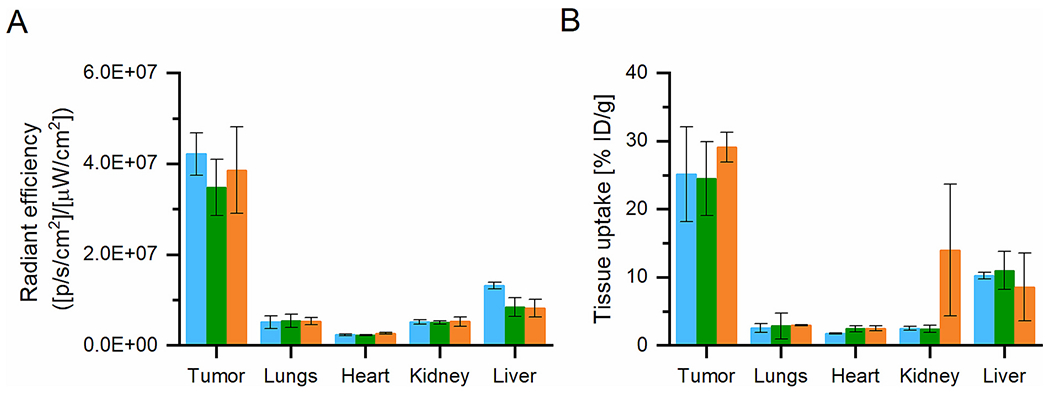
*Ex vivo* analysis of the tumor and major organs 10 days p.i. Images are representative of 4 mice per group: A) Region-of-interest analysis calculated from *ex vivo* images of fluorescence activity retained in tumor and major organs at day 10 p.i. of the SF1 (blue), SF2 (green) and SF4 (orange); B) Percentage of the ID/g of SPet1 (blue), SPet2 (green) and SPet4 (orange) in selected organs 10 days p.i., determined from the PET ROI analysis of tumor and major organs *ex vivo*. (For interpretation of the references to color in this figure legend, the reader is referred to the web version of this article.)

The preferential sustained accumulation of the star polymers SF1, SF2, and SF4 in tumor tissue was proved also by optical imaging *in vivo* (Fig. S7) and *ex vivo* (Figs. 5 and 6A). Analogously to the PET imaging, the inner structure of the star core did not influence tumor accumulation, since the time activity curves of tumor accumulation showed almost identical accumulation in the tumor area for all the star polymer systems (Fig. 5A). Therefore, the tumor accumulation of the tested star carriers is strictly hydrodynamic size dependent.

Moreover, the *ex vivo* imaging (Fig. 5B) was in line with the *in vivo* imaging, confirming an almost identical accumulation of star polymers in 4T1 breast tumors. No significant accumulation was found in the healthy organs by fluorescence imaging (Fig. 6A) or PET imaging (Fig. 6B), and the accumulation in the tumor area was 2.5–10 times higher than that observed in the healthy organs, like for the linear LF1 polymer (Fig. 3).

**Fig. 7.**
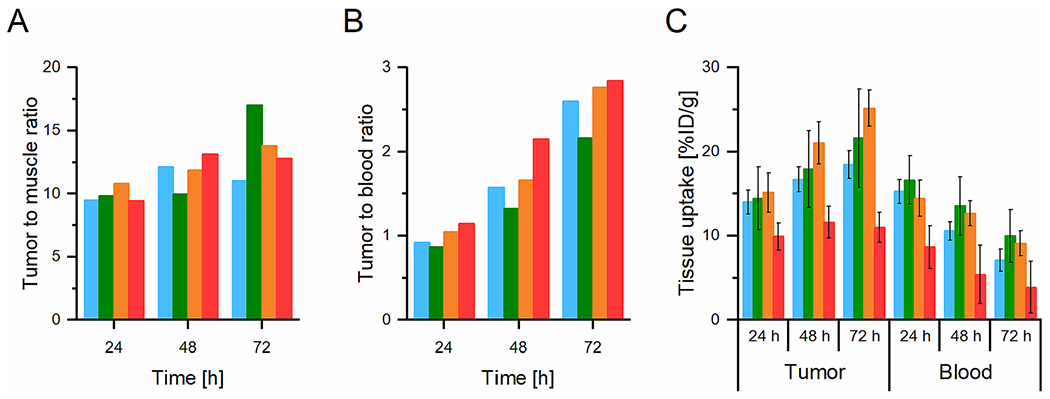
Comparison of *in vivo* biodistribution of the linear and star polymer carriers. A) *In vivo* tumor-to-muscle ratios calculated from quantitative ROI analysis for SPet1 (blue), SPet2 (green), SPet4 (orange), and LPet1 (red) at 24, 48, and 72 h p.i. B) *In vivo* tumor-to-blood ratios calculated from quantitative ROI analysis for SPet1 (blue), SPet2 (green), SPet4 (orange), and LPet1 (red) at 24, 48, and 72 h p.i. C) Comparison of *in vivo* retention of ^89^Zr labeled systems in tumor and blood at 24, 48, and 72 h p.i. Samples: SPet1 (blue), SPet2 (green), SPet4 (orange) or LPet1 (red). (For interpretation of the references to color in this figure legend, the reader is referred to the web version of this article.)

Recently, it was shown [11] that similar HPMA-based star polymers had almost 5 times higher accumulation in solid tumors than the linear HPMA copolymers with broad dispersity and molecular weight of 32,500 g/mol prepared by free radical polymerization (FRP) (maximal molecular weight of FRP polymer ensuring the urine excretion of at least 95% of polymer chains). Indeed, the molecular weight of the linear carrier could be increased up to 40,000 g/mol while guaranteeing the renal elimination of most polymer chains due to utilization of the RAFT polymerization technique for their synthesis. [27] The optimized properties of the linear carrier (LPet1, LF1), *i.e.* increased molecular weight and hydrodynamic size and low dispersity led to improved pharmacokinetics with tumor uptake being 1.5 to 2 times lower than that for the similar mass dose of star polymers (24–72 h p.i.) as determined *via* PET imaging (Fig. 7). PET imaging is an appropriate technique for the head-to-head comparison of the tumor or other tissue accumulation, as it provides the relative part of ID accumulated per g of tissue.

This feature of LPet1 can be explained by the tailored size of the linear carrier allowing prolonged blood circulation in comparison to other polymers prepared previously by FRP and was only approximately two times lower compared to the star polymers at all tested time points (Fig. 7C). There were no significant differences in the tumor-to-blood and tumor-to-muscle ratios among the polymer systems tested (Fig. 7A, B), with the tumor-to-blood ratio increasing with time and peaking at 72 h p.i. Taken together, the pharmacokinetic profiles of star polymers are surprisingly similar to those observed for the linear LPet1 and LF1 copolymers, confirming that the star polymers are unequivocally circulating for a longer time and accumulate to a higher extent in the tumor tissue, with no significant differences regarding the overall distribution and ratios between the tumor and healthy tissues.

#### 3.3.2. Biodistribution of DOX delivered via polymer carriers

PET and fluorescence imaging experiments proved the beneficial enhanced tumor accumulation of both the linear and star polymer nanocarriers compared to off-target tissues. Understanding the pharmacokinetics of polymer-bound drugs is crucial to clarify the relationship between the polymer carrier structure and the treatment efficacy, thus, the total amount of doxorubicin accumulated in various tissues at 6, 12, 24, 48, 72, and 144 h p.i. of the tested conjugates was determined by HPLC to obtain the pharmacokinetic data in the EL4 T cell lymphoma bearing mice.

**Fig. 8.**
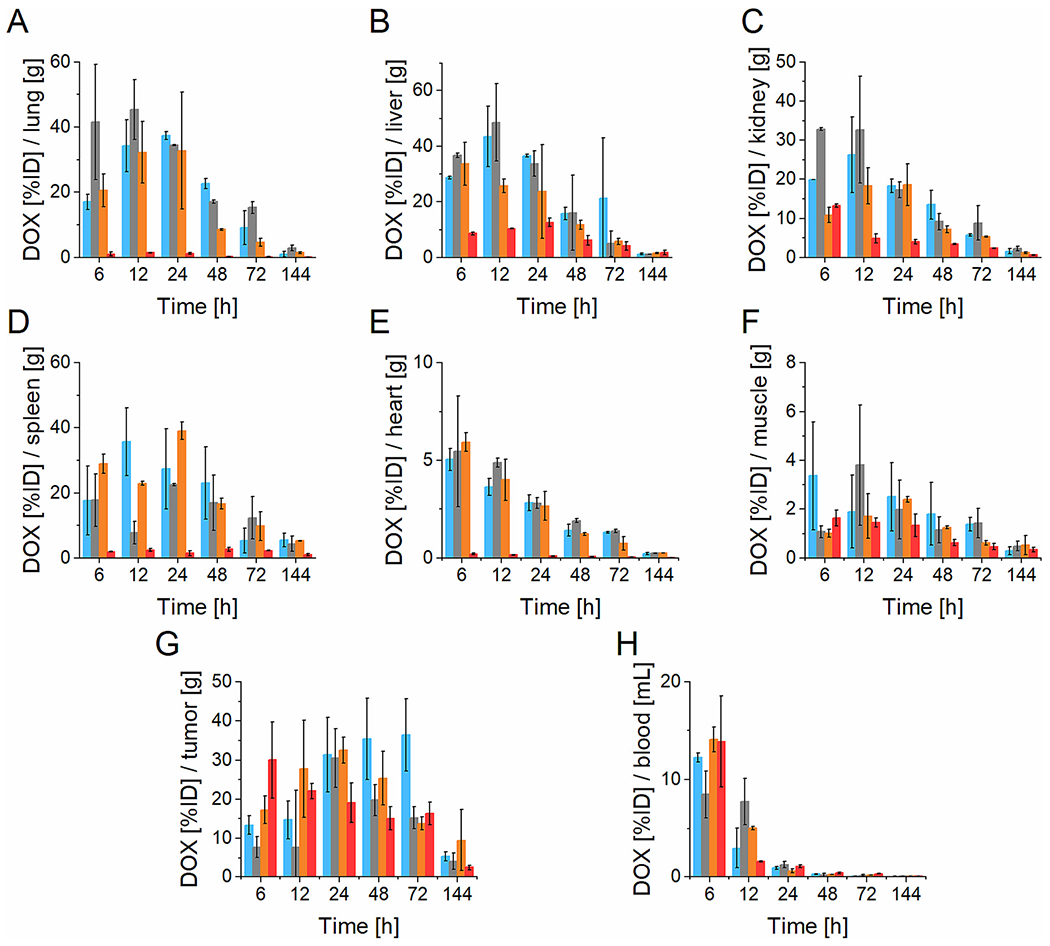
Amount of accumulated DOX in A) lung, B) liver, C) kidney, D) spleen, E) heart, F) muscle, G) tumor and H) blood upon intravenous injection of SC1 (blue), SC3 (grey), SC4 (orange) and LC1 (red) into EL4 tumor-bearing mice. (For interpretation of the references to colour in this figure legend, the reader is referred to the web version of this article.)

The DOX concentration was maximal within 6 or 12 h in all off-target organs and rapidly decreased beyond 24 h (Fig. 8) and corresponded to the high DOX blood concentration. In contrast, the DOX concentration in the tumor remained elevated for at least 72 h p.i. proving the beneficial effect of polymer nanomedicines in drug delivery. Unlike in the biodistribution results, there were significant differences in tumor accumulation among the polymer nanomedicines with different structures. The highest DOX accumulation was observed for the biodegradable SC1 nanomedicine, which maintained the DOX concentration in the tumor at 30% ID of DOX/g of tumor tissue within 24–72 h p.i. Other star conjugates and the linear conjugate reached the maximal DOX concentration in the tumor in a shorter time compared to SC1. Surprisingly, the drug delivery by optimized LC1 nanomedicine was very efficient, especially at shorter time points (6 and 12 h) with the DOX accumulation reaching 20–30% ID of DOX/g of tumor tissue within 24 h and then decreasing to 15% ID of DOX/g of tumor tissue until 72 h p.i. This observation validates the linear LC1 nanomedicine, which has a hydrodynamic size close to the limit of the renal filtration threshold, for prolonged blood circulation and subsequently enhanced tumor accumulation. However, the small hydrodynamic size of LC1 enables rapid extravasation to tumor tissue leading to DOX accumulation in the tumor to peak earlier than the larger star polymer systems. In addition, the hydrodynamic size of the LC1 under the renal elimination limit enables rapid excretion, thereby reducing the overall toxicity. The other star nanomedicines SC3 and SC4 did not show better DOX tumor accumulation than the linear LC1.

Interestingly, all off-target organs except the spleen were almost free of DOX (up to 3% of ID/g) 144 h p.i. of any polymer conjugates regardless of the inner structure and size. The spleen accumulation of DOX 144 h p.i. was still noticeable in the case of star conjugates, whereas the linear conjugate caused almost complete absence of DOX. In addition, the lowest DOX accumulation in healthy tissues at all tested time points was observed upon administration of the LC1 nanomedicine, which showed high potency for selective drug delivery into the tumor.

The biodistribution data (Fig. 8) were further analyzed and the area under the curve (AUC) between 6 and 72 h was calculated for each tissue and expressed as a percentage of the hypothetical AUC (hAUC) for 100 ID%/g of tissue accumulation (Table 6). The star nanomedicines have a high % AUC in tumorous tissue and healthy organs (liver, lung, spleen), whereas the linear LC1 had high tumor accumulation and relatively low healthy organ accumulation.

The overall benefit of the linear nanomedicine is shown by the tumor-to-healthy tissue ratio (Table 7), with the linear nanomedicine exhibiting the highest ratios for all the healthy tissues except blood, where the lower ratio could be ascribed to faster renal elimination and shorter blood circulation compared to the star conjugates. The AUC analysis confirmed the advantage of the linear LC1 with an optimized structure for increased circulation and tumor accumulation.

### 3.4. In vivo antitumor activity

The polymer biodistribution studies showed the potential of various polymer systems to be accumulated at high levels and for a prolonged time in tumor tissue compared to transient low accumulation in off-target tissues. Next, we determined the therapeutic activity of star-like conjugates in two different tumor models, EL4 lymphoma and mammary carcinoma 4T1 with high metastatic potential. The comparative study of *in vivo* activity should include not only the biodistribution study but also the estimation of the maximum tolerated dose (MTD) since the system with high efficiency but a very narrow therapeutic window and high systemic toxicity would not be translated to clinical practice. Systems with sufficient efficacy and low acute, as well as long-term toxicity, are more suitable candidates for further preclinical development and clinical trials.

#### 3.4.1. Maximum tolerated dose (MTD)

The MTD of nondegradable star-like conjugate with a molar mass of 280,000 g/mol and dispersity of 1.5 (10% wt. DOX) was recently recognized as 22.5 mg DOX/kg. [26] Our polymer systems with low dispersity are precisely defined therefore we estimated MTD for the linear polymer at the molecular weight close to the renal excretion limit and in well-defined *star*-like polymers with a hydrolytically stable and non-stable core. Unsurprisingly, the MTD of the linear conjugate LC1 is much higher compared to the non-degradable star-like polymer conjugate, reflecting the ability of the linear carrier to be excreted in the urine rapidly after fulfilling its role in drug delivery, see Table 8. The comparison of the two high molecular weight conjugates is in line with the degradability of the polymer core, as the degradable conjugate was less toxic than the stable conjugate, demonstrating the ability of the polymer chains formed through degradation of the core to be released by renal filtration. The stable SC4 conjugate has an MTD like the star-like polymers published before.

#### 3.4.2. Estimated drug accumulation in tissues based on MTD

**Fig. 9.**
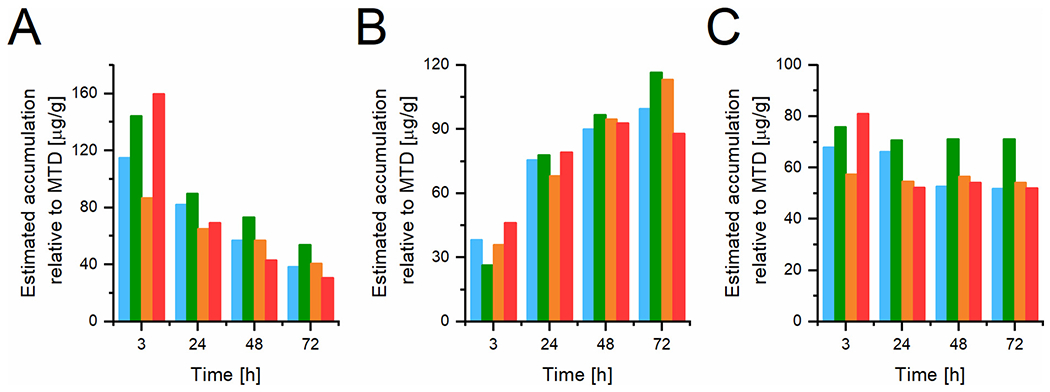
EAD analysis of the PET imaging data after the intravenous injection of SC1 (blue), SC2 (green), SC4 (orange), and LC2 (red) into 4T1 carcinoma-bearing mice: A) blood, B) tumor, C) liver. All the values are expressed as estimated drug in μg /g of tissue. (For interpretation of the references to color in this figure legend, the reader is referred to the web version of this article.)

The estimated maximal drug accumulation for MTD dosing scheme in various tissues was calculated based on the biodistribution data and MTD since this value is difficult to obtain experimentally as the MTD is determined in healthy mice and it often shows some toxicity in tumor-bearing mice. The percentage accumulation (% of ID) was used to calculate the estimated amount of drug (EAD) delivered in the case of MTD dosing, *i.e.*, estimated amount of drug (μg drug/g tissue) = drug (possibly recalculated from carrier accumulation) tissue accumulation (μg /g) multiplied by ratio MTD (mg/kg) to dose used for tissue accumulation experiment (mg/kg). Fig. 9 shows comparable EAD profiles at the MTD dose for all systems studied, with slightly increased tumor accumulation for the linear system after 3 h, and less tumor accumulation only after 72 h compared to the high-molecular-weight star systems. The EAD for the star conjugates is comparable for all tested time points, regardless of their internal structure. The EAD in the blood is comparable for the time points above 24 h and the high EAD for the linear system after 3 h in the blood is most probably caused by low accumulation in healthy organs (lung, spleen, see Fig. 8) causing high retention of the polymer in the bloodstream. Nor the size of star systems above the renal threshold, neither the biodegradability, played any role in the overall tumor accumulation, blood clearance and liver uptake.

**Fig. 10.**
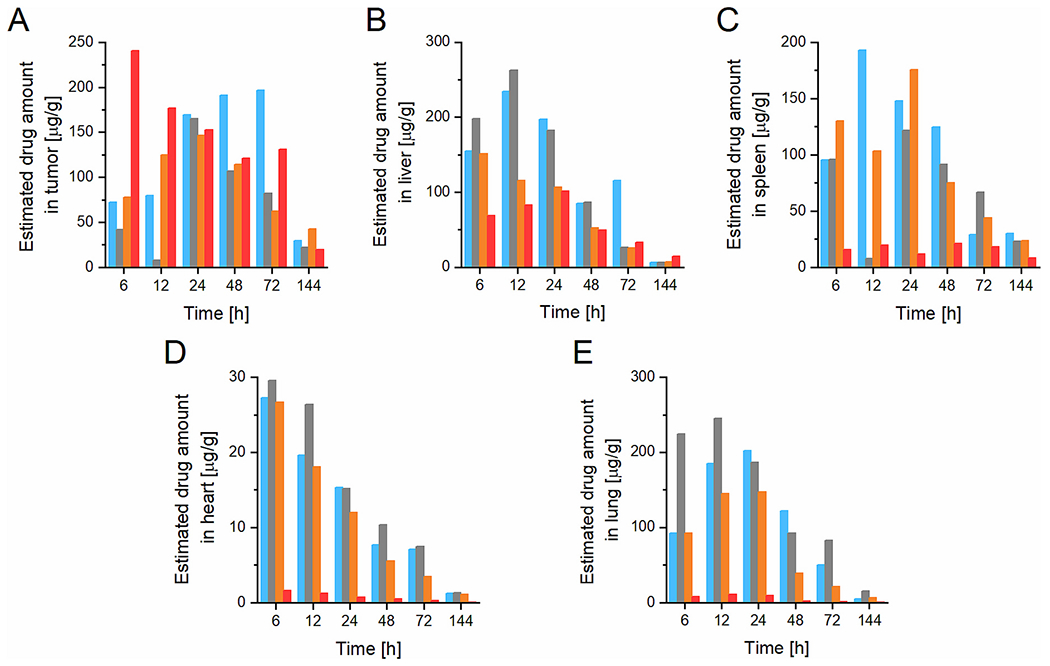
EAD analysis of DOX accumulation data in EL4 lymphoma: A) tumor, B) liver, C) spleen, D) heart, E) lung upon i.v. injection of SC1 (blue), SC3 (grey), SC4 (orange), and LC1 (red) into EL4 tumor-bearing mice. (For interpretation of the references to color in this figure legend, the reader is referred to the web version of this article.)

Regarding the EAD analysis of the DOX accumulation in EL4 lymphoma (Fig. 10), there was a higher EAD in EL4 compared to the 4T1, with a faster onset of accumulation, especially for the linear system, and an overall almost double EAD value compared to 4T1. Thus, the EL4 is more permeable to nanomedicines leading to a higher accumulation due to a more developed EPR effect. Here, the linear conjugate LC1 showed pronounced accumulation peaking at 6 h p.i., subsequently slightly decreasing until 72 h p.i. and dropping to EAD value under 50 μg/g 144 h p.i. when the DOX was almost completely cleared from the tumor. High accumulation, but with a later onset, was found for biodegradable star systems, due to the degradation into polymer fragments with higher extravasation and penetration of tumor tissue than non-degradable star systems.

Importantly, there was a much lower EAD accumulation for DOX in healthy tissues for linear conjugates compared to the star system. Most probably the size around 10 nm did not cause enhanced accumulation in those organs as evidenced by the larger star systems. No significant difference between the liver EAD was found for all the systems.

Taken together, the EAD analysis indicates that LC1 is advantageous for drug accumulation in tumor models with a more pronounced EPR effect compared to star polymer nanomedicines. For the tumor models with a less pronounced EPR effect where the circulation time is the most important parameter increasing the probability of drug accumulation, there were no differences in EAD at MTD dosing for star-like nanomedicines.

#### 3.4.3. Therapeutic activity

**Fig. 11.**
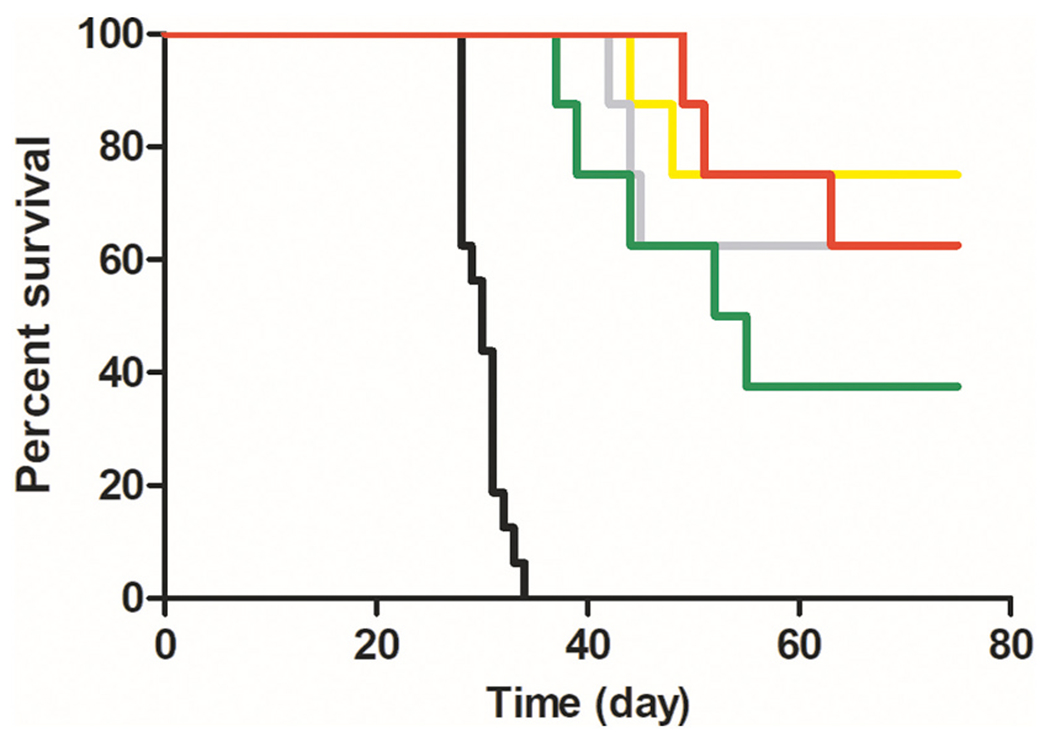
Anticancer activity of DOX-bearing conjugates in EL4 tumor-bearing mice. C57BL/6 mice (*n* = 8) were s.c. injected on day 0 with 2 × 10^5^ EL4 cells. Conjugates were i.v. administered to mice in one dose on day 8. Control mice were left untreated. Survival of experimental mice was recorded. 1 × 7.5 mg/kg DOX equivalent was injected, SC1 (yellow), SC3 (grey), SC4 (green), untreated control (black), and 1 × 15 mg/kg DOX equivalent was injected, LC1 (red). (For interpretation of the references to color in this figure legend, the reader is referred to the web version of this article.)

The preliminary therapeutic activity of the novel star-like systems in the EL4 lymphoma model has been described in our previous study. [21] Herein, we aimed to compare the therapeutic activity of the star nanomedicines with the linear systems with a tailored structure and hydrodynamic size in EL4 lymphoma and 4T1 mammary carcinoma. EL4 lymphoma is an EPR-positive tumor model that is relatively sensitive to DOX treatment and all the high-molecular-weight conjugates SC1, SC3, and SC4 proved significant anticancer activity even at a low single dose of 7.5 mg DOX/kg, equivalent to 33.5 (SC4) or 27.5% (SC1 – SC3) of MTD (Fig. 11). The survival time of EL4 tumor-bearing mice was prolonged significantly, with the dendron-type SC1 being the most effective, followed by the bis-MPA-based SC3, and the stable SC4 nanomedicine cured three out of eight animals. Thus, the bisMPA conjugate is an excellent drug carrier with some treatment benefits most probably reflecting the degradation of this system, thus better extravasation and tissue penetration of the linear grafts after degradation. *Star*-like polymer systems based on the biodegradable bisMPA core are more effective for drug delivery to tumors and treatment than *star*-like polymers with the same physicochemical characteristics and hydrolytically stable core. Importantly, LC1 exhibited excellent therapeutic activity at 15 mg DOX eq./kg, equivalent to 37% of MTD, curing five out of eight animals. Thus, the treatment efficacy of the linear nanomedicine is comparable to the bio-degradable star nanomedicines discussed above and all the polymer nanomedicines cured at least five out of eight animals at a dose below 40% of MTD.

Moreover, the cured animals were resistant to a second challenge with EL4 tumor cells administered 77 days after the first transplantation (data not shown). This is in line with our previous work demonstrating the development of anti-tumor immunity in mice with experimental tumors and cured with HPMA-based polymer conjugates carrying cytostatic drugs. [28–30]

The 4T1 carcinoma is a mouse model significantly resistant to chemotherapy. The treatment of 4T1 with free DOX (3 mg/kg, equivalent to 50% of MTD [31]) had a negligible effect on tumor growth. Even 10-dose scheme of free DOX had no effect on the 4T1 tumor growth inhibition. [32] Progression of 4T1 tumors is accompanied by a dramatic increase in white blood cell count (WBC) [33,34], consisting mostly of immature neutrophil-like and monocyte-like myeloid cells referred to as myeloid-derived suppressor cells (MDSC). MDSCs contribute significantly to the immunosuppressive nature of tumor microenvironment in 4T1 model.

**Fig. 12.**
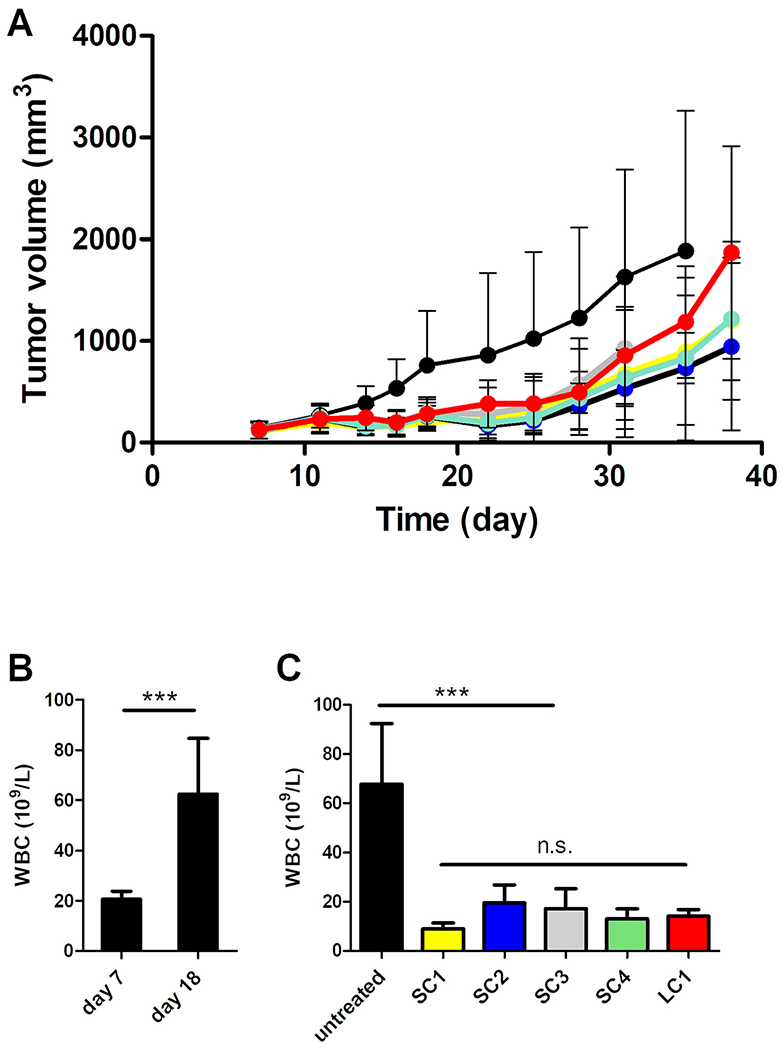
A) Anticancer activity of polymer-DOX conjugates in 4T1 tumor-bearing mice. BALB/c mice (n = 8) were s.c. injected on day 0 with 2 × 10^5^ 4T1 cells. Conjugates were i.v. administered to mice in 2 doses on days 8 and 11. Control mice were left untreated. A) Tumor volume was recorded SC1 (yellow), SC2 (blue), SC3 (grey), SC4 (green), LC1 (red), untreated (black). B) Blood cell analysis was performed on day 18 post-tumor cell inoculation and the WBC count in peripheral blood was determined using a hemoanalyzer. WBC counts in untreated mice on days 7 and 18, mean and SD are depicted. C) WBC counts in all groups on day 18. (For interpretation of the references to color in this figure legend, the reader is referred to the web version of this article.)

The therapeutic activity of the biodegradable polymer conjugates with a bisMPA core reduced primary tumor growth (Fig. 12A). Moreover, the WBC counts (Fig. 12 B C) and neutrophils (Fig. S8) determined 1 week after the second dose were significantly reduced but animals did not survive longer than the untreated controls (Supporting Information, Fig. S9).

Regarding systemic toxicity, there was a transient decrease in body weight exceeding 15% of the initial value in the groups treated with two doses, each equivalent to 40% of the estimated MTD, of SC1 and SC4 (Fig. S9B). Three early deaths without significant body weight loss occurred in the groups treated with SC2, SC4, and LC1, which could be attributed to systemic toxicity (Fig. S9A), so a longer interval between doses may be safer. However, the fast tumor progression in the 4T1 model may represent a risk of decreased therapeutic efficacy due to the delayed second dose. In summary, two doses of all polymer nanomedicines (each equivalent to 40% of MTD) inhibited 4T1 tumor growth mainly in the initial phase after injection but the growth inhibition was limited and tumors began to grow again. The designed nanomedicines are more effective than treatment with free DOX since the injection of nanomedicines inhibited tumor growth. There was no significant difference between the linear and *star*-like polymer nanomedicines at an equitoxic dose.

### 3.5. Nanomedicine evaluation

**Fig. 13.**
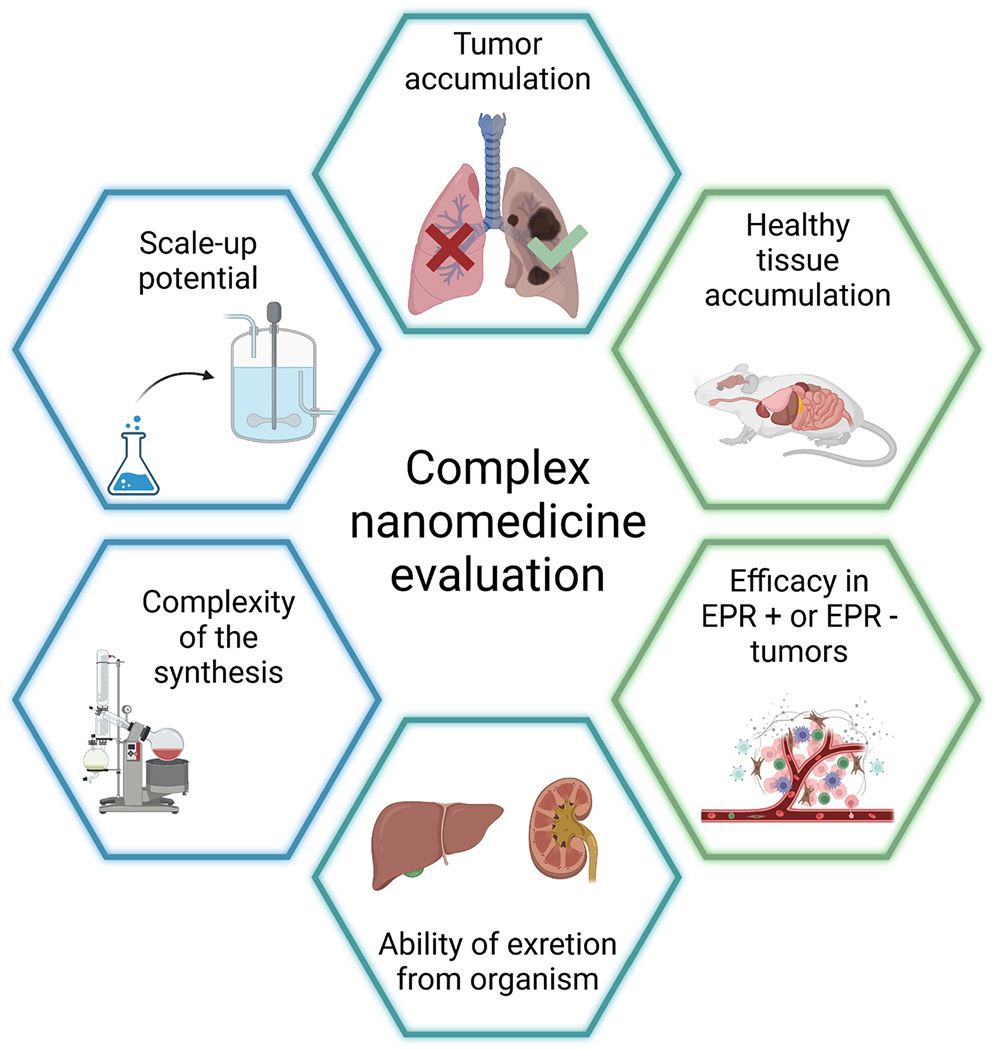
Overall scheme of the complex nanomedicine evaluation approach. Key parameters for evaluation from the chemical and biological point of view.

The overall suitability of individual nanomedicines for clinical translation was evaluated using seven parameters determining the properties and advantages of the studied nanomedicines (Table 9) including i) complexity of synthesis in terms of the number of reaction steps and procedures; ii) scale-up potential of the synthetic technology; iii) accumulation in the tumor; iv) accumulation in healthy tissue; v) efficacy of the anti-cancer treatment on the well-vascularized tumor model (EPR+ tumors); vi) efficacy of the anti-cancer treatment on the poorly vascularized tumor model (EPR− tumors); vii) elimination of the carrier. This tool was designed for universal multi-parametric comparison of diverse nanomedicines since many aspects of potential therapeutics play an important role in further clinical translation. Overall graphical scheme of the complex nanomedicine evaluation is shown in Fig. 13.

A comparison of the linear LC1, star-like biodegradable SC1, and control star non-biodegradable SC4 using the tool in Table 9 revealed that LC1 and SC1 exhibited comparable treatment efficacy and tumor biodistribution to the control SC4 (Table 10). However, the biodegradability of SC1 brought some advantages, especially in the elimination rate, tumor accumulation, and treatment efficacy compared to SC4. Surprisingly, the linear LC1 exhibited a considerably safer biodistribution and elimination profile while having a comparable anticancer activity to SC1. However, the scale-up potential of SC1 was poor due to the more complex synthesis. Importantly, the linear nanomedicine with tailored physicochemical properties for prolonged circulation and enhanced tumor accumulation is the most promising nanomedicine for the forthcoming pre-clinical testing, see Table 10.

## 4. Conclusion

The tailored nanomedicines based on a biodegradable star polymer or a tuned linear polymer structure were compared using three biodistribution techniques showing both the polymer carrier and model drug biodistribution. Interestingly, if the biodistribution and therapeutic effectiveness data were related to the MTD of individual nanomedicines, the most promising results were achieved by the linear polymer carrier designed for prolonged circulation and a biodegradable *star*-shaped system with a relatively fast degradation of the structure into smaller polymer grafts. Both nanomedicines exhibited comparable potential as effective anti-cancer therapy but the linear nanomedicine was considered safer due to its very low uptake into healthy tissues and organs. Furthermore, the linear nanomedicine with a tailored structure enabling prolonged blood circulation, tumor accumulation, and subsequent renal elimination is the most suitable candidate for further clinical translation.

## Supplementary Material

SI

## Figures and Tables

**Table 1:** Characterization of synthesized linear polymer precursors.

Precursors	*M*_n_^a^ [g/mol]	Ð^a^	NHNH_2_ [mol%]	*R*_h_^b^ [nm]	F_TT_^c^
LP1	36,600	1.1	5.7	4.5 ± 0.2	–
LP2	38,500	1.1	5.6	4.7 ± 0.7	0.9

a determined by SEC.

b determined in PBS at 25 °C by a viscometric detector.

c TT end group content was determined spectrophotometrically at 305 nm.

**Table 2:** Characterization of synthesized linear polymer conjugate.

Conjugate	*M*_n_^a^ [g/mol]	Ð^a^	*R*_h_^b^ [nm]	DOX [wt%]
LC1	40,600	1.1	4.9 ± 0.2	9.1

a Determined by SEC.

b Determined in PBS at 25 °C by a viscometric detector.

**Table 3:** Characterization of synthesized *star*-like polymer precursors.

Precursors	Core	Core Generation	Z^c^	*M*_n_^a^ [g/mol]	Ð^a^	*R*_h_^b^ [nm]
SP1	MPA dendron-NH_2_	4	7.1	274,000	1.1	10.4 ± 0.8
SP2	MPA dendrimer-NH_2_	3	7.3	282,000	1.1	11.5 ± 0.8
SP3	MPA dendrimer-N_3_	3	7.6	293,000	1.1	11.7 ± 0.5
SP4	PAMAM	3	7.3	280,000	1.1	11.3 ± 0.4

a Determined by SEC.

b Determined in PBS at 25 °C by a viscometric detector.

c Number of polymer arms conjugated on the dendritic core.

**Table 4:** Characterization of synthesized *star*-like polymer-drug conjugates.

Conjugate	Biodegradability	Precursor	*M*_n_^a^ [g/mol]	Ð^a^	*R*_h_^b^ [nm]	DOX [wt %]
SC1	Fast	SP1	300,000	1.2	9.3 ± 1.0	9.3
SC2	Fast	SP2	270,000	1.1	10.3 ± 0.8	9.4
SC3	Slow	SP3	310,000	1.3	10.5 ± 0.7	9.9
SC4	Stable	SP4	278,000	1.2	11.0 ± 0.5	9.5

a Determined by SEC.

b Determined in PBS at 25 °C by a viscometric detector.

**Table 5:** Characterization of Dy676 or DFO labeled linear and *star*-like polymer conjugates.

Conjugate	Biodegradability	Precursor	DFO [mol.%]	Dy676 [mol.%]
LPet1 [27]	–	LP1	3.20	–
LF1 [27]	–	LP1	–	0.20
SPet1	Fast	SP1	0.35	–
SF1	Fast	SP1	–	0.95
SPet2	Fast	SP2	0.36	–
SF2	Fast	SP2	–	0.77
SPet4	Stable	SP4	0.58	–
SF4	Stable	SP4	–	0.78

**Table 6:** The AUC of polymer nanomedicines expressed as a percentage of the hAUC.

Sample	Tumor (% hAUC)	Liver (% hAUC)	Muscle (% hAUC)	Kidney (% hAUC)	Blood (% hAUC)	Lung (% hAUC)	Spleen (% hAUC)
SC1	30.7	26.9	2.0	15.4	1.3	25.6	22.5
SC3	22.9	24.2	1.8	15.6	1.5	26.5	17.5
SC4	25.2	16.8	1.5	11.6	1.6	18.2	22.9
LC1	18.1	8.3	1.0	4.1	1.4	0.7	2.2

**Table 7:** Tumor to healthy organ ratios calculated from the data in Table 6.

Sample	Tumor/liver ratio	Tumor/muscle ratio	Tumor/kidney ratio	Tumor/blood ratio	Tumor/lung ratio	Tumor/spleen ratio
SC1	1.1	15.3	2.0	20.1	1.2	1.4
SC3	0.9	12.8	1.5	16.0	0.9	1.3
SC4	1.5	16.8	2.2	10.4	1.4	1.1
LC1	2.2	18.9	4.5	6.1	24.3	8.2

**Table 8:** Maximum tolerated dose of selected nanosystems in BALB/c mice.

Conjugate	Biodegradability	*M*_n_^a^ [g/mol]	Ð^a^	MTD [mg DOX/kg]
LC1	–	40,600	1.1	40
SC1	Fast	300,000	1.2	27
SC2	Fast	270,000	1.1	27
SC4	Stable	277,000	1.2	22.5

a Determined by SEC.

**Table 9:** Definition of the scale of individual parameters for the evaluation of nanomedicines.

Rating scale	Synthesis simplicity	Scale up possibility	Tumor accumulation % AUC between 6 and 72 h	Healthy tissue accumulation % AUC between 6 and 72 h (average of liver, spleen, kidney, and lung)	Treatment efficacy, EPR+ tumors	Treatment efficacy, EPR− tumors	Elimination of carrier (more than 90% of ID)
1	Multistep synthesis, purification needed	Not scalable as the individual steps are difficult to reproduce	Very low tumor accumulation, below 5% AUC	Very high accumulation, above 25% AUC	Very low effectivity at 50% MTD dosing, small reduction in tumor growth	Very low effectivity at 50% MTD dosing, small reduction in tumor growth	Long-term retention, more than 2 months
2	Multistep synthesis, short purification needed	Scalable with problems – some steps are difficult to reproduce	Low tumor accumulation, 5–10% AUC	High accumulation, 15–25% AUC	Low effectivity at 50% MTD dosing, some reduction in tumor growth	Low effectivity at 50% MTD dosing, some reduction in tumor growth	Mainly *via* the hepato-biliary route, less than 2 months
3	A low multistep synthesis, purification needed	Scalable but some steps require an advanced approach to be scalable	Medium tumor accumulation, 10–15% AUC	Medium accumulation, 10–15% AUC	Medium effectivity at 50% MTD dosing, less than 50% LTS	Medium effectivity at 50% MTD dosing, less than 50% LTS	*Via* the urine and hepato-biliary route, less than 1 month
4	Easy and reproducible synthesis, few steps, purification and separation needed	Easily scalable but some steps need an advanced approach for scale-up	High tumor accumulation, 15–25% AUC	Low accumulation, 5–10% AUC	High effectivity at 50% MTD dosing, more than 50% LTS	High effectivity at 50% MTD dosing, more than 50% LTS	*Via* urine in two weeks
5	Easy and reproducible synthesis, low number of steps, easy purification and separation	Easily scalable	Very high tumor accumulation, above 25% AUC	Very low accumulation, below 5% AUC	Very high effectivity at 50% MTD dosing, more than 75% LTS	Very high effectivity at 50% MTD dosing, more than 75% LTS	NA

LTS – long-term survival.

**Table 10:** Evaluation of the nanomedicines.

Sample	Synthesis simplicity	Scale up possibility	Tumor accumulation % AUC between 6 and 72 h	Healthy tissue accumulation % AUC between 6 and 72 h (average of liver, spleen, kidney, and lung)	Treatment efficacy, EPR+ tumors	Treatment efficacy, EPR− tumors	Elimination	Overall rating
LC1	4	4	4	5	5	2	4	28
SC1	2	3	5	2	5	2	3	22
SC4	2	3	5	2	4	2	1	19

## Data Availability

Data will be made available on request.
