# Supplementary material for: Evaluation of linear versus star-like polymer anti-cancer nanomedicines in mouse models
Source: J Control Release. Author manuscript; Available in PMC 2024 Jan 1. (PMC9892306; doi:10.1016/j.jconrel.2022.11.060)
Supplement: SI [file NIHMS1857034-supplement-SI.docx]

Supporting information

Evaluation of linear versus star-like polymer anti-cancer nanomedicines
in mouse models

*Libor Kostka^a†^, Lenka Kotrchová^a†^, Eva Randárová^a^, Carolina A. Ferreira^b^, Iva Malátová^c^, Hye Jin Lee^d^, Aeli P. Olson^e^, Jonathan W. Engle^e^, Marek Kovář^c^, Weibo Cai^d ,e^, Milada Šírová^c^, Tomáš Etrych^a^**

*^†^*Authors contributed equally to the publication.

^a^*Institute of Macromolecular Chemistry CAS, Department of Biomedical Polymers*

*Heyrovského nám. 2, 162 06 Prague 6, Czech Republic*

^b^ *Department of Biomedical Engineering, University of Wisconsin-Madison, Madison, WI, United States*

^c^ *Institute of Microbiology CAS, Laboratory of Tumor Immunology, Vídeňská 1083, 142 20 Prague 4, Czech Republic*

^d^ *Department of Pharmaceutical Sciences, University of Wisconsin-Madison, Madison, WI, United States*

^e^*Departments of Radiology and Medical Physics, University of Wisconsin-Madison, Madison, WI, United States*

*Corresponding author: etrych@imc.cas.cz*

Keywords: drug delivery, cancer, polymeric carriers, HPMA, biodistribution, positron emission tomography

The authors dedicate this paper to Prof. Blanka Říhová, DSc. on the occasion of her 80th birthday.

# 2 Experimental Section/Methods

## **2.1 Materials**

Doxorubicin hydrochloride (DOX) was purchased from Meiji Seika, Japan. Dendritic cores, PFD-G3-TMP-azide (24 end groups) dendrimer, PFD-G3-TMP-Ammonium (24 end groups), and PFD-G4-acetylene-ammonium (16 end groups) dendron were obtained from Polymer Factory (Sweden). PAMAM dendrimer (G3), CuBr, 8-quinolinol, and DIPEA were purchased from Merck-Sigma Aldrich (Czech Republic). Azo initiator V-70 was obtained from Wako Chemicals (Japan). P-SCN-Bn-Deferoxamine (DFO) was purchased from Macrocyclics™ (USA) and Dy676 was purchased from Dyomics GmbH (Germany). All solvents were anhydrous or dried and distilled. The synthesis of monomers *N*-(2-hydroxypropyl) methacrylamide(HPMA) and *N*-(*tert*-butoxycarbonyl)-*N*’-(6-(methacryloylamino) hexanoyl)hydrazine (MaAhNHNH-Boc) were described previously.[1, 2] The CTA-AIBN, S-2-cyano-2-propyl-S-ethyl trithiocarbonate for the synthesis of linear conjugates was synthesized as described by Ishitake et. al. [3] Thiazolidine-2-thione functional CTA 2-cyano-5-oxo-5-(2-thioxo-1,3-thiazolidin-3-yl)pentane-2-yl ethyl carbontrithioate (CTA-TT) was synthesized as described previously. [4]

## **2.5 Characterization of polymers**

The weight-average molecular weight *M*_w_, number-average molecular weight *M*_n_ and dispersity *Ð* of polymer precursors and conjugates were measured using by SEC on an HPLC Shimadzu system equipped with an SPDM20A photodiode array detector (Shimadzu, Japan), differential refractometer (Optilab®rEX), and multiangle light scattering (DAWN HELLEOS II) detectors (both from Wyatt Technology Co., USA). The polymer precursors and conjugates were characterized on a Superose6 or Superose12 column with 0.1M PBS (pH 7.4) mobile phase. The size and conformation of polymer chains were determined by the online differential viscometer ViscoStarIII (Wyatt Technology Co.). The hydrodynamic radius (*R*_h_) was measured by dynamic light scattering (DLS) (Nano-ZS, Malvern) in 0.15 M NaCl solution at a polymer concentration of 5 mg mL^-1^ at 25°C. The amount of doxorubicin and fluorescence dye Dy676 was determined spectroscopically (DOX: ε_488_ = 11 200 L mol^-1^ cm^-1^, methanol; Dy676: ε_676_ = 110 000 L mol^-1^ cm^-1^, methanol).

## **2.6 Hydrolytic stability**

The hydrolytic stability of SP1, SP2, and SP3 (3 mg mL^-1^) was determined in 0.15 M phosphate buffer at pH 5.0 or 7.4 at 37°C. The degradation rates were estimated by HPLC with an SEC column TSKgel4000SWxL.

## **2.7 Animals and tumor models**

Inbred female C57BL/6 and BALB/c mice were obtained from the animal facility of the Institute of Physiology CAS, Prague, Czech Republic, and were bred under conventional conditions, food, and water *ad libitum*. The protocol was approved by the Institutional Animal Care and Use of the Academy of Sciences of the Czech Republic and conducted in compliance with local and European guidelines.

## **2.8 Toxicity *in vivo***

To determine the maximum tolerated dose (MTD), female BALB/c mice were injected with an increasing single dose of the conjugates. Body weight and signs of deteriorated physical condition (hunched posture, coat condition, reduction of food and water intake) were recorded daily or every other day for at least three weeks (n=3 per conjugate/dose). The results were summarized from two independent experiments and the limit value for systemic toxicity was set as a body weight loss of less than 15% of the initial body weight. Mice injected with PBS were used as controls.

## **2.9 *In vivo* biodistribution studies**

*PET imaging*

LPet1, SPet1, SPet2, and SPet4 polymer conjugates were radiolabeled with ^89^Zr according to previously published studies.[4] PET imaging was performed in 4T1 tumor-bearing mice after intravenous injection of 5-10 MBq of the radiolabeled compounds using an InveonmicroPET/microCT rodent model scanner (Siemens Medical Solutions USA, Inc) as previously described [5] 0.5, 4, 24, 48, 72, 120, 168 and 240 h post-injection (p.i.). The images were reconstructed using a maximum a posteriori (MAP) algorithm with no attenuation or scatter correction. Decay-corrected whole-body images were used to calculate the total radioactivity and are presented as the percentage of injected dose (%ID). All whole-body imaging studies were conducted according to the protocol approved by the University of Wisconsin Institutional Animal Care and Use Committee.

*Optical imaging*

In vivo fluorescence studies of Dy676 conjugated polymers LF1, SF1, SF2, and SF4 were also conducted in 4T1 tumor-bearing mice (n = 4) and images were captured on a PerkinElmer IVIS system using 675/720 nm excitation/emission filters 0.5, 4, 24, 48, 72, 120, 168, and 240 h post intravenous administration of the compounds. After the last scan time point, animals were euthanized and 4T1 tumors, hearts, spleens, lungs, kidneys, and livers were harvested and imaged ex vivo to validate the in vivo findings., ROIs were drawn on the explanted tumor and organs using vendor software and the total signal intensity presented in units of radiant efficiency (p s^−1^ cm^−2^ sr^−1^)/(μW cm^−2^) within the ROI was used for subsequent semi-quantitative analysis.

## **2.10 Determination of DOX content in tissues**

The DOX content of mice tissues was determined as described previously.[4] Briefly, female C57BL/6 mice were transplanted with EL4 lymphoma cells and injected with polymer conjugates on day 8. Heparinized blood, heart liver, kidneys, lung, spleen, muscle, and tumor tissue samples were collected 6, 12, 24, 48, 72, and 144 h after treatment. The tissues were excised, weighed, and homogenized in PBS, and the DOX content (i.e., the sum of free and polymer-bound DOX) was determined after quantitative acid hydrolysis by HPLC.

## **2.11 *In vivo* treatment studies, blood analysis**

A murine EL4 T cell lymphoma model was established for *in vivo* experiments. Female C57BL/6 mice (*H-2b*) were subcutaneously (s.c.) transplanted at day zero with 1.10^5^ EL4 cells. Then, the mice were treated intravenously (i.v.) with the star DOX conjugates SC1, SC3, and SC4 on day 8, when the diameter of the tumors was 5–8 mm, at a dose of 7.5 mg DOX/kg i.v. The LC1 conjugate was injected at the same time at a dose of 15 mg DOX/kg i.v. Tumor growth and survival time were monitored. The tumor volume was calculated as V = a∙b^2^/ 2, where a = longer diameter, and b = shorter diameter. Mice with fully regressed tumors (long-term survivors, LTS) were re-transplanted with the same number of EL4 lymphoma cells and left untreated.

Murine 4T1 breast carcinoma tumors were established in female BALB/c mice by injecting 2.10^5^ 4T1 cells s.c. on day zero. The mice were treated with two i.v. doses of conjugates, each equivalent to 40% MTD on days 8 and 11, and the tumor growth and survival were monitored. Body weight was measured as a quantitative parameter of systemic toxicity. Moreover, a blood count was performed on day 18 using a veterinary blood analyzer (Mindray BC-5000 Vet, China) to obtain the white blood cell (WBC) and neutrophil count as an additional quantifiable parameter of tumor progression. For the analysis, a small sample of non-coagulable peripheral blood (30 μL) was collected from the tail vein using heparin-coated plastic capillaries intended for blood gas sampling (Webers, Germany). The analysis was performed immediately after blood collection.

## **2.12 Statistical analysis**

Statistical analysis of tumor growth and survival time was conducted using two-way ANOVA and log-rank (Mantel-Cox) tests with GraphPad Prism software. A p < 0.05 was considered statistically significant, p < 0.001 as highly significant

# Supporting information figures





Figure S1: A) *In vitro* optical imaging and B) quantification of samples containing a corresponding amount of single dose received per mouse (demonstrating the use of same amount of dye for each sample). C) ^89^Zr labelling yields as a function of incubation time at 37°C, pH 7–8 (n=3)


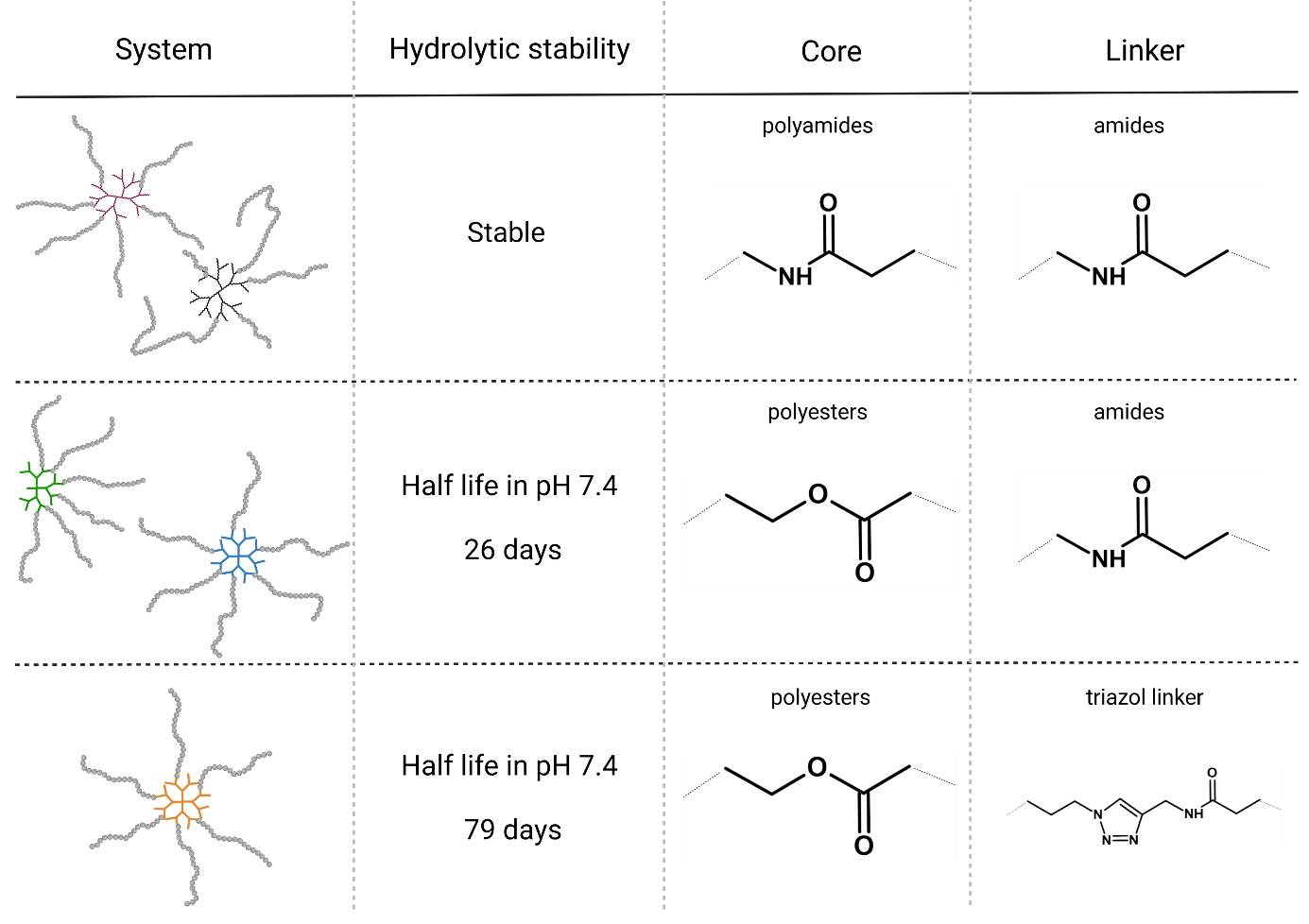


Figure S2: Schematic overview of developed systems with described chemical properties.


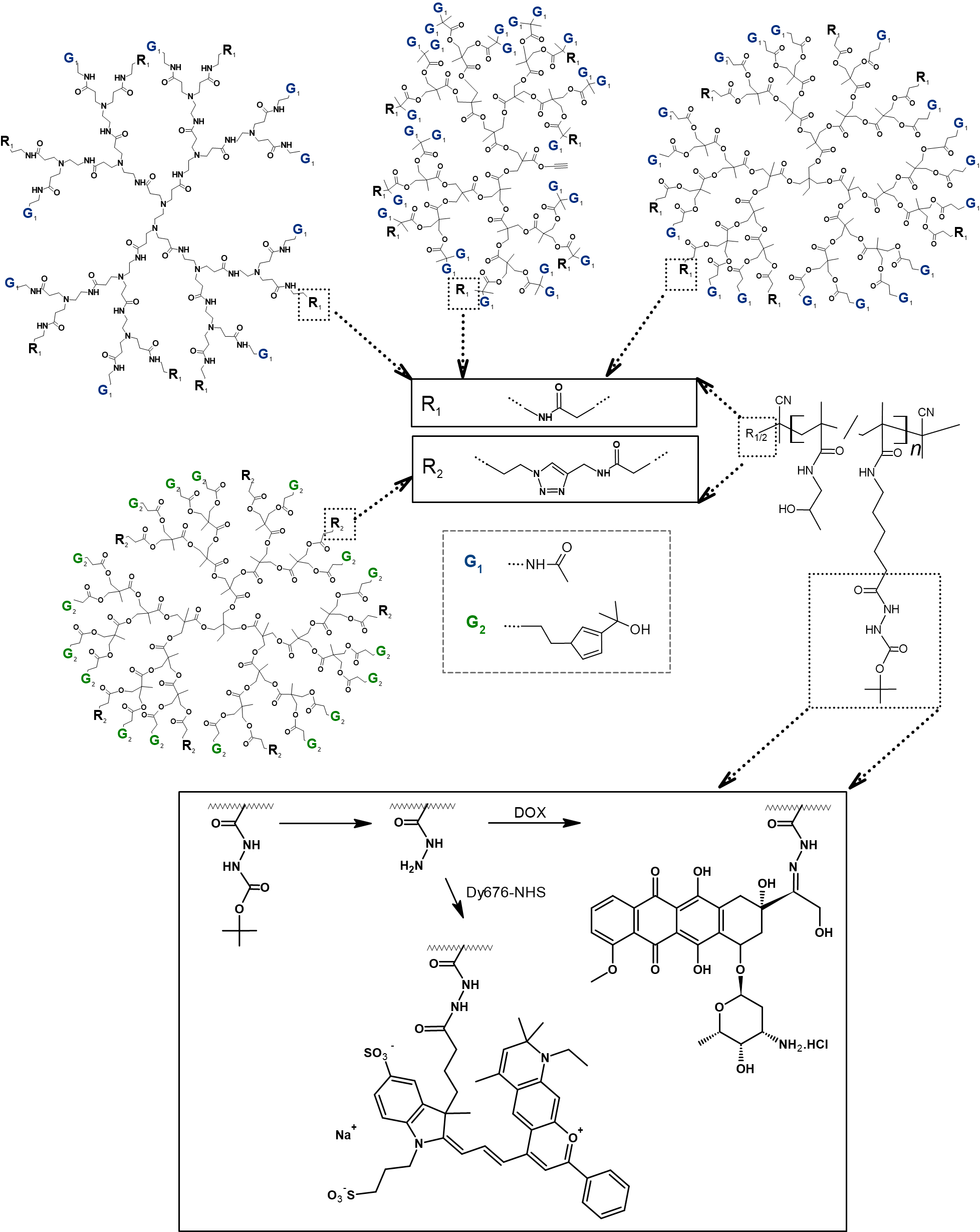


Figure S3: Schematic structures of developed star systems.





Figure S4: Hydrolytic stability in PBS with pH 7.4 (square) and pH 5.0 (circle) of polymer precursor SP1 (green) and SP2 (blue) and SP3 (orange).


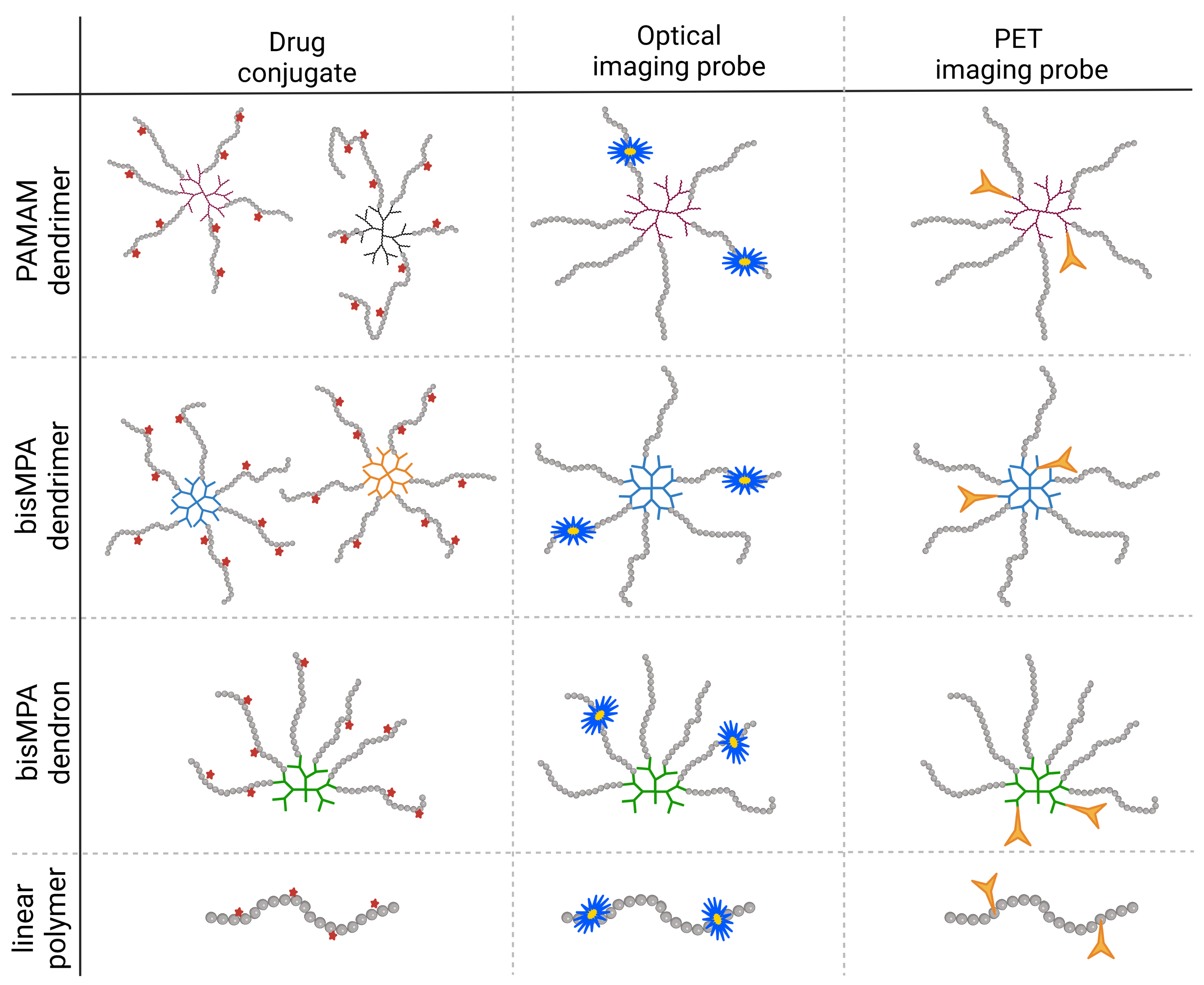


Figure S5: Schematic representation of the polymer systems used in this study for therapeutic purposes and also for biodistribution studies.



Figure S6: Serial maximum intensity projection (MIP) in vivo PET images of 4T1 tumor-bearing mice injected with ^89^Zr-SPet1 ^89^Zr-SPet2 or ^89^Zr-SPet4, at different time points post-injection. The green circles indicate the 4T1 tumors.





Figure S7: Serial in vivo optical imaging of 4T1 tumor-bearing mice injected with SF1, SF2 and SF4 at different time points post-injection. Images are representative of 4 mice per group.


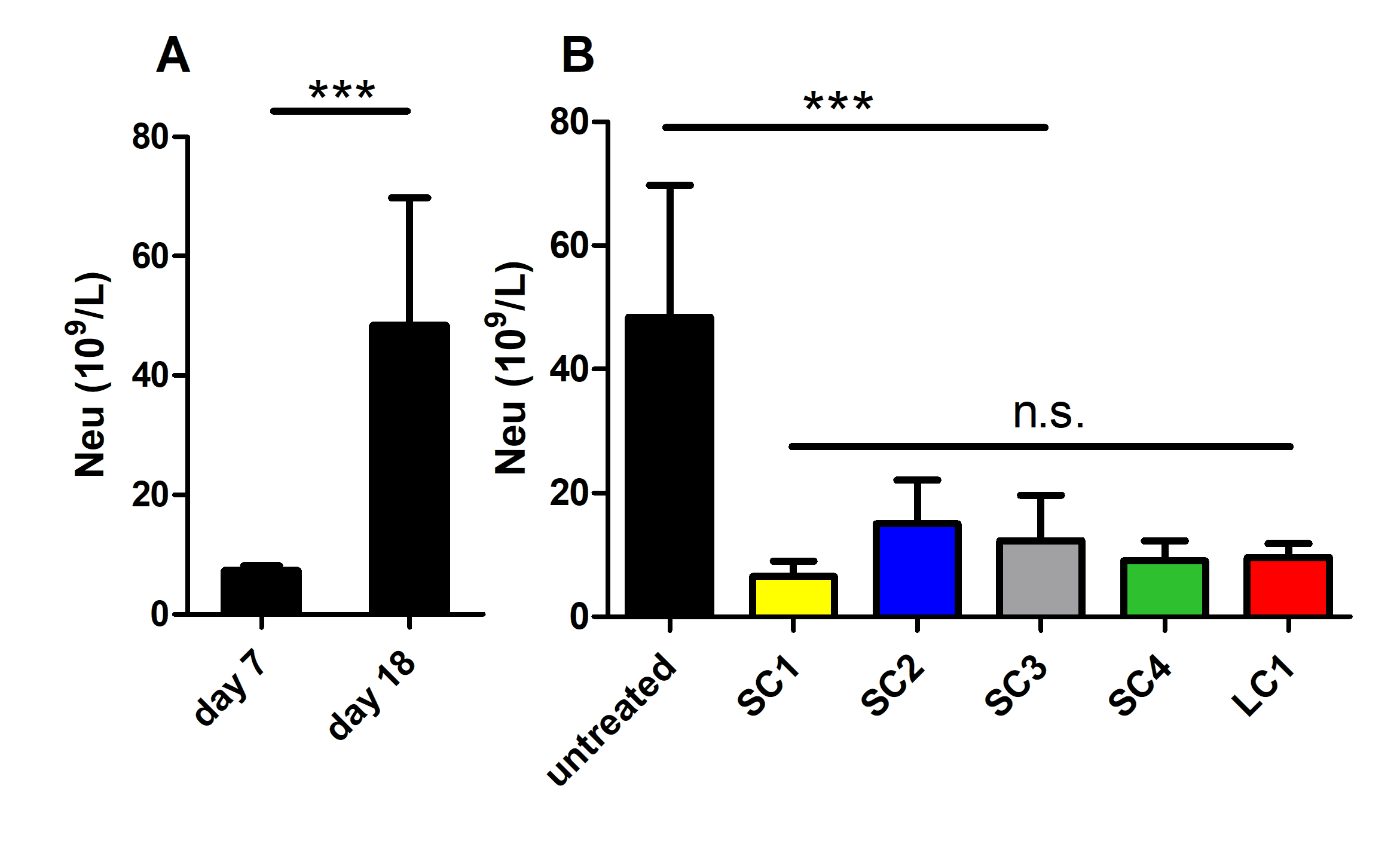


Figure S8: Anticancer activity in the 4T1-bearing mice treated as described in Figure 13. A) Neutrophil counts in peripheral blood determined via hemoanalyzer in untreated mice on days 7 and 18, mean and SD are depicted. B) Neutrophil counts in all groups determined on day 18.


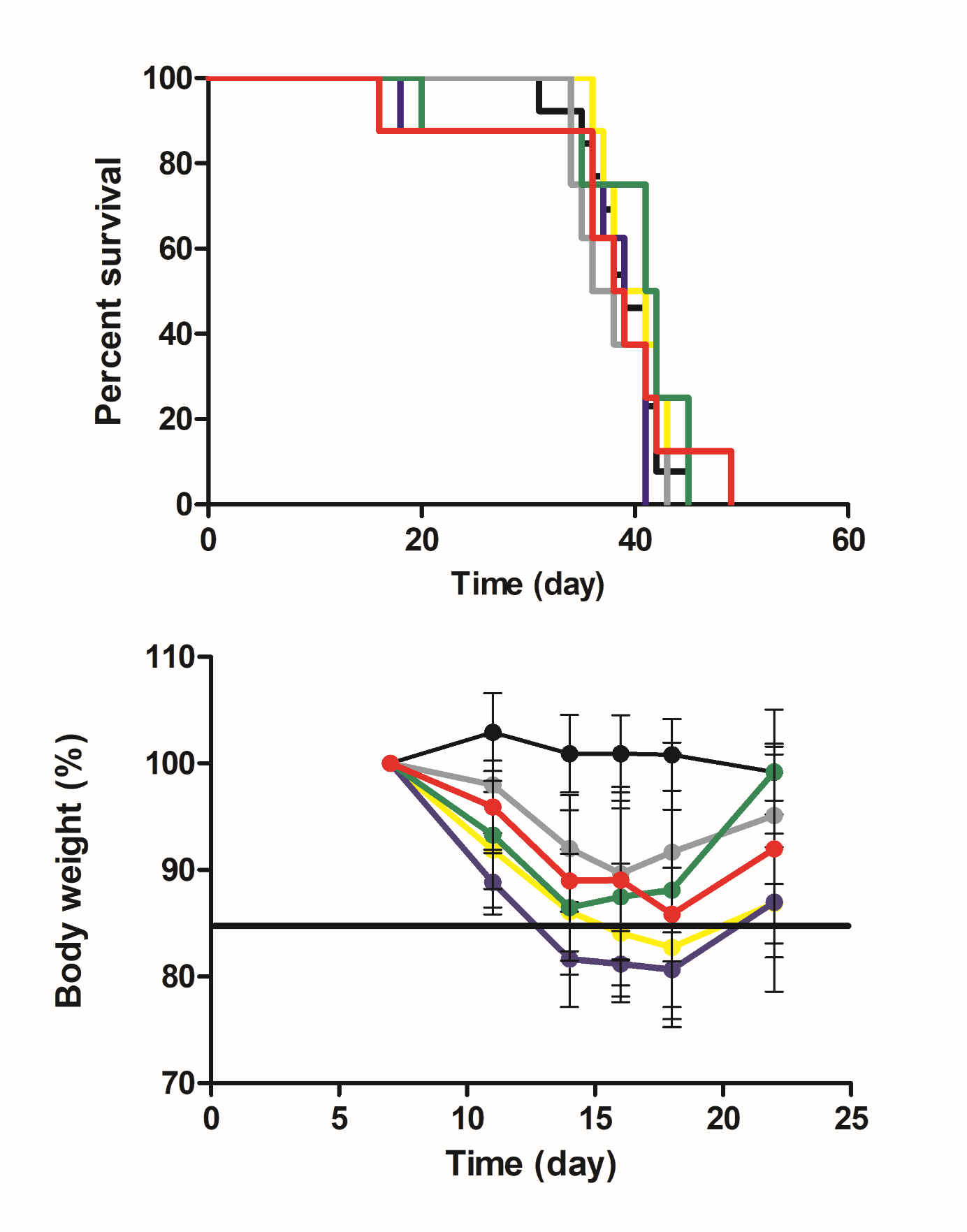


Figure S9: Survival of 4T1-bearing BALB/c mice treated with conjugates carrying DOX (tumor growth and WBC counts depicted in Fig. 6). The mice (n=8) were s.c. injected on day 0 with 2x10^5^ 4T1 cells. Conjugates were i.v. administered to mice in two doses on days 8 and 11. Control mice were left untreated. A) Survival of mice was recorded. SC1 (yellow), SC2 (blue), SC3 (grey), SC4 (green), LC1 (red), untreated (black). B) Body weight as a measurable parameter of systemic toxicity of the treatment was monitored. The cut off value was body weight decrease exceeding 15 % of the initial value.

**References**

[1] K. Ulbrich, V. Subr, J. Strohalm, D. Plocova, M. Jelinkova, B. Rihova, Polymeric drugs based on conjugates of synthetic and natural macromolecules I. Synthesis and physico-chemical characterisation, J. Control. Release, 64 (2000) 63-79.

[2] K. Ulbrich, T. Etrych, P. Chytil, M. Jelinkova, B. Rihova, Antibody-targeted polymer-doxorubicin conjugates with pH-controlled activation, J. Drug Target., 12 (2004) 477-489.

[3] K. Ishitake, K. Satoh, M. Kamigaito, Y. Okamoto, Stereogradient Polymers Formed by Controlled/Living Radical Polymerization of Bulky Methacrylate Monomers, Angew. Chem. Int. Ed., 48 (2009) 1991-1994.

[4] L. Kostka, L. Kotrchova, V. Subr, A. Libanska, C.A. Ferreira, I. Malatova, H.J. Lee, T.E. Barnhart, J.W. Engle, W. Cai, M. Sirova, T. Etrych, HPMA-based star polymer biomaterials with tuneable structure and biodegradability tailored for advanced drug delivery to solid tumours, Biomaterials, 235 (2020) 119728.

[5] E. Koziolová, S. Goel, P. Chytil, O. Janoušková, T.E. Barnhart, W. Cai, T. Etrych, A tumor-targeted polymer theranostics platform for positron emission tomography and fluorescence imaging, Nanoscale, 9 (2017) 10906-10918.
